# Prehospital plasma transfusion versus standard of care following traumatic injury: a review of the systematic reviews and a meta-analysis

**DOI:** 10.1007/s00068-025-03033-z

**Published:** 2025-11-27

**Authors:** Ayman El-Menyar, Sandro Rizoli, Mashhood Naduvilekandy, Ammar Al-Hassani, Fernando Spencer Netto, Mohammad Asim, Naushad A. Khan, Basar Cander, Sagar Galwankar, Lukasz Szarpak, Ruben Peralta, Hassan Al-Thani

**Affiliations:** 1 Clinical Research, Trauma and Vascular Surgery, Hamad Medical Corporation, Doha, Qatar; 2 Department of Medicine, Weill Cornell Medical School, Doha, Qatar; 3 Department of Surgery, Trauma Surgery, Hamad Medical Corporation, Doha, Qatar; 4 Department of Emergency Medicine, Bezmialem Vakif University, Fatih, Istanbul, Turkey; 5 Department of Emergency, Emergency Medicine Residency Program, Florida State University College of Medicine, Sarasota Memorial Hospital, Sarasota, FL USA; 6 Technology Transfer Center, The Paul II Catholic University of Lublin, Lublin, Poland; 7 Department of Surgery, Universidad Nacional Pedro Henriquez Urena, Santo Domingo, 10100 Dominican Republic

**Keywords:** Prehospital, Plasma, Blood product transfusion, Efficacy, Safety, Outcomes, Meta-analysis, Systematic review, Trauma

## Abstract

**Background:**

Individual studies suggest that administering prehospital blood products such as plasma to injured patients is feasible, may lower mortality, and improve coagulation. By compiling all existing evidence, we aimed to investigate whether prehospital plasma (PHP) transfusion can be safely administered and improve the clinical outcomes of trauma patients.

**Methods:**

A systematic review (SR) and meta-analysis were conducted in accordance with the PRISMA guidelines to assess the effectiveness and safety of PHP transfusion compared to the standard of care in trauma patients. A literature search (2012 and 2024) was performed in PubMed, MEDLINE, EMBASE, and the Cochrane Library using the terms: “plasma resuscitation,” “prehospital plasma,” “prehospital blood components,” “emergency transfusion,” “trauma hemorrhage management,” “lyophilized plasma,” “freeze-dried plasma " “LyoPlas,” FlyPlas,” and “thawed fresh frozen plasma .“Studies focused on pediatric patients, in-hospital settings, feasibility only, or non-plasma interventions were excluded. Primary outcomes included early (24 hours) and late (28 or 30 days) mortality, and secondary outcomes included 24-hour transfusion units, vasopressor use, multiple organ failure, transfusion reaction, acute lung injury, and sepsis. The quality of studies was assessed using the Newcastle-Ottawa Scale and the Cochrane Risk of Bias tool. The review was registered with PROSPERO. Sensitivity analyses were performed, excluding small studies with high variance and studies with combined blood products.

**Results:**

Twelve studies comprising 3,193 trauma patients (1,579 intervention and 1,614 control arm) and seven SRs were included. There was no significant difference between the PHP and control groups for early and late mortality; however, the sensitivity analysis favored the PHP transfusion for 24-hour mortality. Without statistical significance, the total 24-hour volume of RBC units and vasopressor use was lower in the PHP group than in the controls. There was no significant difference between the PHP transfusion and control groups for the incidence of organ failure, adverse events, transfusion reactions, and sepsis. Observational studies were mostly of good quality, with two studies showing a moderate risk of bias. In contrast, RCTs had some concerns but were generally at a low risk for most domains.

**Conclusion:**

The overall pooled analysis revealed no significant benefit to PHP transfusion in trauma patients; however, sensitivity analyses showed a significant association of PHP and lower 24-hour mortality. PHP did not significantly decrease vasopressor use or late mortality; however, it may reduce the total use of RBCs in the first 24 h. Regarding safety, the review findings should be interpreted cautiously. Umbrella review was not conducted due to the heterogeneity and inconsistent inclusion criteria and outcomes. Further studies are needed to address the inconsistency in the existing evidence and determine whether PHP transfusion should be recommended for trauma patients with clearer and standardized endpoints and adverse event reporting.

**Supplementary Information:**

The online version contains supplementary material available at 10.1007/s00068-025-03033-z.

## Introduction

 Traumatic hemorrhage remains the leading cause of potentially preventable trauma-related deaths, accounting for over 40% of all such fatalities, with nearly half occurring before hospital arrival [1]. A common complication of traumatic hemorrhage is trauma-induced coagulopathy (TIC), observed in 20–50% of patients upon hospital arrival. It is independently associated with a five-fold increase in mortality [2–4]. Early administration of blood products, especially plasma, in the prehospital setting may reduce mortality, correct TIC, and prevent later complications of bleeding. Two types of plasma have been investigated for prehospital use: thawed fresh frozen plasma (FFP) and freeze-dried or lyophilized plasma (FDP), including single-donor LyoPlas^®^ and pooled-donor FlyPlas^®^. Plasma is currently the only blood product approved in a stable, dehydrated form for use in prehospital settings. It can be easily rehydrated within minutes. Thawed and lyophilized plasma are comparable in quality—containing similar levels of fibrinogen, factor XI, protein C, and antithrombin—although lyophilized plasma has a slightly lower factor VIII content (25% reduction) and a more alkaline pH [4]. However, FDP’s ability to be stored at room temperature (up to 25 °C) for a year makes it particularly suitable for field use, despite being more expensive than FFP. Although whole blood — which contains both plasma and red blood cells — is increasingly explored in trauma resuscitation, its short shelf-life, storage limitations, and cross-matching requirements hinder its prehospital application compared to plasma, especially FDP [4].

Plasma transfusion restores depleted intravascular volume, replaces clotting factors, corrects hyperfibrinolysis, raises serum albumin levels, preserves endothelial function and physiological pH, and may reduce 30-day mortality by 10% [2, 5–7]. FFP is stored at −18 °C and requires thawing before use, while FDP is stored at room temperature (up to 25 °C) with a shelf life of 15 months, showing no significant decrease in clotting factor levels [4]. FDP is pathogen-inactivated and does not require cross-matching. To assess fibrinogen concentration at 45 min after randomization and within 6 h of injury, Garrigue et al. randomly assigned patients to lyophilized FLyPlas or FFP. The study showed that FLyPlas had a higher fibrinogen concentration, a better prothrombin time ratio, and improved coagulation parameters, including factor V and factor II, compared to FFP [8].

A strong biological rationale suggests that early plasma transfusion in severely injured patients is necessary to prevent and treat TIC [6, 9]. The prevailing damage control resuscitation (DCR) includes rapid hemorrhage control, permissive hypotension (if not contraindicated), minimization of crystalloids, prevention of coagulopathy, and, as early as possible, transfusion of red blood cells (pRBC) and plasma [10]. To reduce hemorrhage-related complications, including overt TIC, irreversible shock, and uncontrolled inflammatory response, DCR is ideally initiated early in the prehospital phase [11–13]. However, logistical reasons are the main reason preventing the early use of blood products outside the hospital. Many centers, mainly in the USA, have thawed or liquid plasma, along with red blood cells, for administration in both the ED and prehospital settings [14].

Early prehospital transfusion studies from military and civilian hospitals have demonstrated that prehospital plasma (PHP) and packed red blood cell (pRBC) administration are feasible and associated with improved coagulation upon hospital arrival and better outcomes [2, 10, 11, 15–18]. Despite the solid rationale favoring PHP, the existing literature remains inconclusive [5, 19], arguably due to the challenge of studying the effect of plasma versus that of other confounders, such as injury severity, transport time, and coagulation modifiers, compounded by the difficulties of storing plasma.

We reviewed the existing systematic reviews (SRs) to provide a comprehensive and up-to-date synthesis of the conflicting evidence [4, 20–25]. We performed a thorough, up-to-date systematic review (SR) and meta-analysis on the effectiveness and safety of PHP transfusion in trauma. The aim is to test the hypothesis that PHP transfusion can be administered safely and effectively and improve the clinical outcomes of trauma patients.

## Methods

**Fig. 1:**
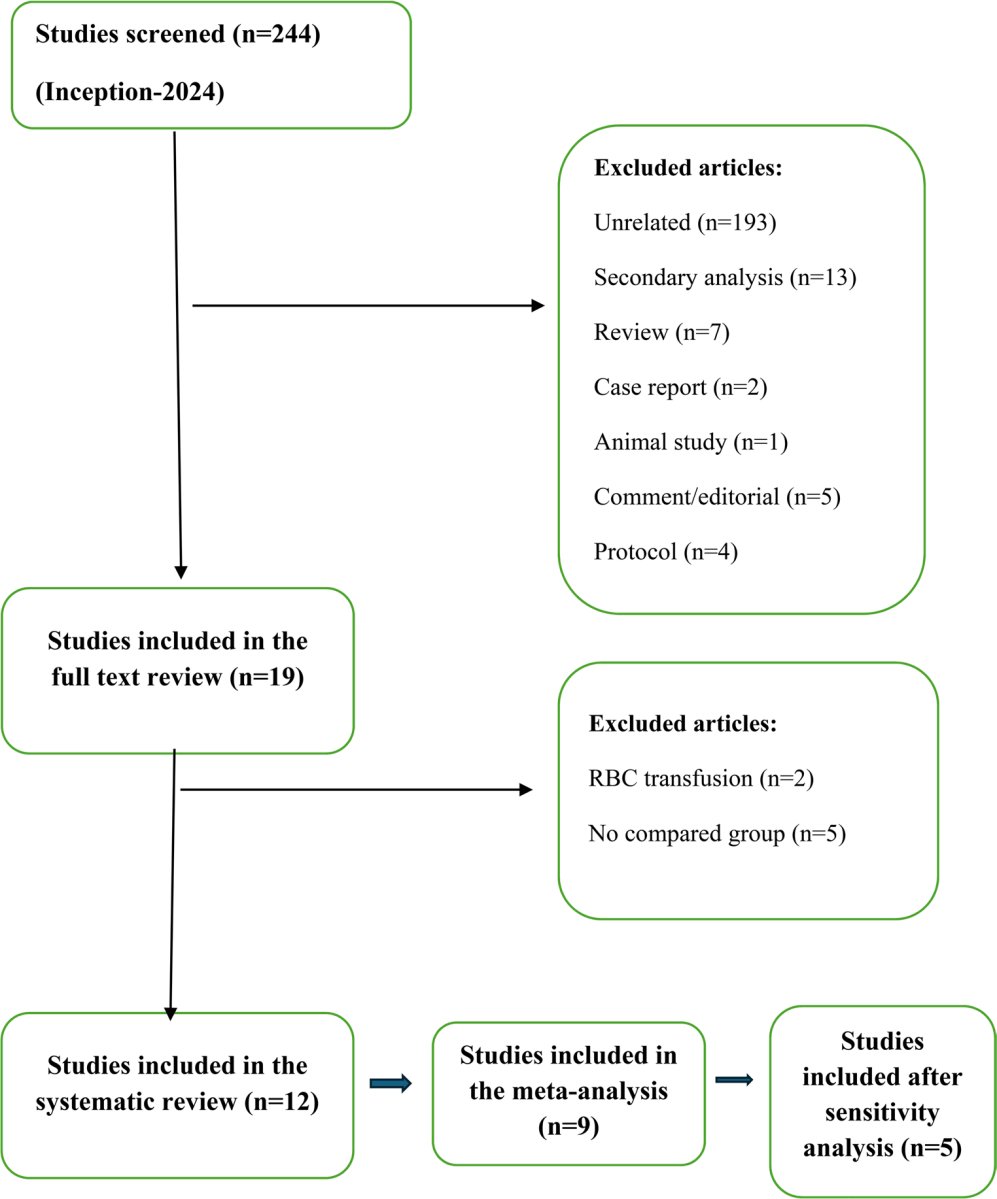
PRISMA flowchart of included studies

The present SR and meta-analysis were conducted in accordance with the Preferred Reporting Items or Systematic Reviews and Meta-Analyses (PRISMA) guidelines (Fig. 1). The review employed a systematic search strategy and adhered to predefined inclusion and exclusion criteria. The study was registered with the International Prospective Register of Systematic Reviews (PROSPERO) under the ID number CRD42024599358.

### Literature search

The literature search included studies published between January 2012 and December 2024, utilizing electronic databases such as PubMed, MEDLINE, EMBASE, and the Cochrane Library. Two reviewers (MNK and AMR) independently conducted the database searches and assessed the eligibility of all studies identified in the initial search. Full-text articles of the selected studies were retrieved and screened after deduplication using the web tool (Rayyan.ai) to confirm their suitability for inclusion in the meta-analysis. Any disagreements were resolved through consultation with a third author (AEM). Key search terms included “plasma resuscitation,” “prehospital plasma,” “prehospital blood components,” “emergency transfusion,” “prehospital fluid therapy,” “trauma hemorrhage management,” “Lyophilized plasma,” “freeze-dried plasma,” and “LyoPlas” (Supplementary Table 1). Additionally, references from original articles and prior meta-analyses were manually reviewed to identify relevant citations. In addition, we reviewed the contemporary related SRs.

##  Outcomes

Primary outcomes included early (24-hour) and late mortality (28 or 30 days), and secondary outcomes included 24-hour transfusion units, vasopressor use, multiple organ failure (MOF), transfusion reaction, acute lung injury (ALI), and sepsis.

### Eligibility criteria

We included studies focusing on adult trauma patients requiring PHP transfusion (fresh frozen or lyophilized plasma), and the comparison group received standard care with resuscitation fluids like normal saline, pRBC, and other solutions without plasma. The primary outcomes assessed were all-cause mortality (24 h, 28 days, or 30 days (in-hospital or during follow-up). Secondary outcomes included the total volume of plasma and RBC transfusions within 24 h, vasopressor use, hemostatic effect (INR, PT, or TGA), MOF, ALI, transfusion reactions, and sepsis. Studies focused on pediatric patients, in-hospital settings, or non-plasma interventions were excluded. Additionally, case reports, experimental studies, commentaries, editorials, and study protocols were excluded. Feasibility-only or non-plasma intervention studies were excluded.

### Data extraction

Two reviewers (MNK and AEM) independently extracted relevant data from the included studies using a predefined template in an Excel spreadsheet. Disagreements were resolved through discussion with a third author (AEM). The main characteristics of the studies included the author, year of publication, study location, design, settings, duration, study arms or interventions, age, sex, number of patients by group, indication for PHP, outcomes, and important comments. A meta-analysis was conducted to evaluate both the primary and secondary outcomes.

### Risk of bias assessment and quality of evidence

The included studies were assessed for risk of bias using the Newcastle-Ottawa Scale (NOS) for observational studies and the Cochrane Risk of Bias-2 (ROB-2) assessment tool for randomized controlled trials (RCTs). Two reviewers (MNK, AMR) independently assessed the studies for potential biases related to selection, deviations from the intended interventions, reporting missing outcome data, measurement of the outcome, and overall study quality. The quality of evidence was assessed using the GRADE (Grades of Recommendation, Assessment, Development, and Evaluation) tool by the same two authors.

### Statistical analysis

Binary outcomes were analyzed using both random-effects and fixed-effects methods, depending on the level of between-study variance (heterogeneity). Heterogeneity between studies was assessed using the I² statistic, which quantifies the proportion of total variation across studies that is due to heterogeneity rather than chance. I² values of 0–25% were considered low, 25–50% moderate, and > 50% substantial heterogeneity. In our analysis, we applied a fixed-effects model when heterogeneity was negligible (I² ≤ 25%), and a random-effects model (DerSimonian and Laird method) when heterogeneity was greater than 25%. We chose this threshold to adopt a conservative approach, acknowledging that even moderate heterogeneity in trauma research may reflect meaningful differences in study design, patient population, or interventions. Random-effects models provide more robust estimates under such conditions, as they account for variability in the underlying effect size across studies. For dichotomous outcomes, pooled risk ratios (RRs) with 95% confidence intervals (CIs) were calculated, whereas for continuous outcomes, the mean difference (MD) with 95% CI was used.

For continuous outcomes, some included studies reported medians with interquartile ranges (IQRs) or ranges, rather than means with standard deviations (SDs). To allow inclusion of these studies in the pooled analysis, we converted medians to means and estimated SDs using the validated method proposed by Wan et al. [26]. This approach has been widely used in meta-analyses and shown to yield accurate approximations, particularly when sample sizes are not small and the data distribution is not highly skewed. Studies in which plasma was not administered to all patients in the intervention group were excluded from the meta-analysis.

Sensitivity analysis was performed after excluding small studies with high variance. For sensitivity analyses, studies were considered small if either treatment or control group included fewer than 10 patients. Studies were considered highly heterogeneous if their reported effect sizes and confidence intervals diverged markedly from the pooled estimate, or if their design/population differed substantially from those of the other included studies. Sensitivity analysis was also conducted to distinguish the effects of pRBC combined with plasma from those of plasma alone within the intervention group. Statistical analysis was performed using Python libraries, including Pandas, SciPy, and Matplotlib, for data manipulation, hypothesis testing, and visualization (Python 3.0).

## Results

### Study selection

The literature search criteria identified 244 publications. After screening the titles and abstracts, 193 unrelated publications were excluded (Fig. 1). Thirteen studies underwent secondary analyses, primarily using data from two RCTs on PHP transfusion in trauma patients [5, 19].

### Characteristics of the included studies

The SR included twelve studies [2, 5, 13, 15, 17–19, 27–31] and 3,193 trauma patients (1,579 in the intervention and 1,614 in the control arm). The baseline characteristics of the studies are summarized in Tables 1 and 2. The studies included four RCTs [13, 19, 27, 28], one cluster RCT [5], three prospective studies [2, 17, 29], and four retrospective studies [15, 18, 30, 31]. Six studies were multicenter, and one was in Afghanistan (military). Air (helicopter) was the primary mode of transportation in seven studies [5, 15, 17, 18, 28, 29, 31], while two studies reported on ground transportation alone [19, 27]. All studies had a male predominance in the intervention (67–98%) and control groups (60–98%). The median age of the intervention group ranged from 33 to 54 years.

**Table 1:** Baseline characteristics of studies included in the systematic review and meta-analysis

Author/Year/duration	Study design/country	Age and sex per group	*N* of patients per group	MOT	Indication	Outcomes	Comment
Sperry 2018 [5] May 2014 -Oct 2017 PAMPer	Cluster **RCT**, **Multicenter**, USA	Median 46 vs. 44 yrs. Male 74% vs. 71%	271 Standard care resuscitation (Crystalloid or pRBC) vs. 230 **thawed** plasma (2U)	**Air**	Injured adults with SBP < 90 mm Hg and HR > 108 or any episode SBP < 70 mm Hg	30-day mortality (34% vs. 24%). 24-hour mortality (22% vs. 14%) & in-hospital mortality (32.5% vs. 22.2%). No differences (MOF, infection, and transfusion-related events)	**Subanalysis**: Prehospital **pRBC** (113/172 vs. 59/172 (30-day mortality 34.5% vs. 28.8%). **Prolonged prehospital transport time** (30-day mortality 37% vs. 23.6%).
Moore 2018 [19] April 2014 - March 2017 COMBAT	**RCT**, **Single**-center, USA (ITT and as-treated analysis)	33 vs. 32.5 yrs. Male 80% vs. 85%	65 **thawed** plasma (2U) vs. 60 control pts (NLS).	**Ground**	Adults (age > 18) with SBP ≤ 70 mm Hg or 71–90 mm Hg and HR 108 thought to be due to acute blood loss	28-day mortality (15% in the plasma vs. 10% in the control, *p* = 0∙37). **No significant differences** in safety outcomes and adverse events.	In plasma group, 21 (32%) received 2 units during transport; 24 (37%) received 1 unit during transport and the second unit in the ED; and 20 (31%) started the first plasma unit during transport but it was completed and followed by the second unit in the ED. There was a consistent lack of differences > > the study was stopped for futility. PH coagulopathy was rare. PHP does not improve outcomes when given within 30 min during transportation. PHP could be beneficial in difficult environments or longer transport time.
Jost 2022 [27] April 2016- Sep 2019* PREHO-PLYO	**RCT**, **Multicenter**, France	33.6 vs. 36.6 yrs. Male 77% vs. 87%	66 controls (up to 1000 Crystalloid vs. 68 **Lyo** plasma (up to 4 U) pts	**Ground**	Severe trauma ≥ 18 yrs at high risk for hemorrhagic shock and associated coagulopathy (If SBP < 70 mm Hg or a SI > 1.1	6-hr mortality (3% vs. 4.4%, *p* = 0.6), 24-hr mortality (9.1% vs. 13.2%, *p* = 0.4), 28-day mortality (15.2 vs. 17.6%, *p* = 0.7).	**PHP** (lyo) was **not** associated with significant differences in INR values, MOF and sepsis; it was feasible and safe. Tranexamic acid was used (91% vs. 84%).
Holocomb 2017 [17] Jan - Nov 2015 PROHS	**Prospective**, pragmatic, Multicenter, USA	39 vs. 48 yrs, Male 73% vs. 67%	After **propensity score matching**: 66 (no **PHT** but crystalloid) pts vs. 43 (received **PHT**: **thawed** plasma and/or RBCs) pts	**Air**	At least one (1) HR > 120, (2) SBP ≤ 90 mmHg, (3) penetrating truncal injury, (4) tourniquet applied, 5) pelvic binder applied, 6) intubated prehospital, or 7) received blood products during Transport. Age > 15 yrs	3-hr mortality (OR 0.74, *p* = 0.60), 24-hr mortality (OR 0.74, *p* = 0.58), or 30-day mortality (OR 0.85, *p* = 0.75)	Of patients receiving PHT, 24% received only plasma, 7% received only RBCs and 69% received both. The authors stated that obtaining 80% power to detect a 4% absolute reduction in all-cause mortality would require 3996 for 3 h, 4970 for 24 h, and 6672 patients for a 30-day survival study.
Tucker 2023 [29] Oct 2018 - Oct 2020	**Prospective**, **Multicenter**, UK	36 vs. 43 vs. 35.6 yrs. Male 80.5% vs. 75.5% vs. 83.8%	**Retrospective**: 223 RBCs (up to 4 U) vs. **prospective**: 391 2U R BCs + 2U **thawed** or Lyoplas Plasma (RCB + Plasma) vs. 295 Red cells and plasma (RCP)* *	**Air**	Injured started receiving or received at least one type of blood component or product [if clinical suspicion of (or confirmed) hemorrhage and b) SBP < 90 mmHg (at any time)]. All ages included.	24-hour mortality (47.5% vs. 36.1% vs. 40.2%), 30-day mortality (53.8% vs. 49.1% vs. 50.1%). RCP and RBC + Plasma were associated with lower 24-hour mortality compared to RBC alone (aOR 0.69 [*p* = 0.012] and 0.60 [*p* = 0.11], respectively) Ω.	The lower odds of death for RBC + Plasma and RCP vs. RBC were shown with **penetrating** injury (**not** with blunt injury) (aOR 0.22 [*p* = 0.001] and 0.39 [*p* = 0.006], respectively). There was no association between RCP or RBC + Plasma vs. RBC with 30-day survival
Shlaifer et al. 2018 [30] Jan 2006-December 2016	**Retrospective**, matched cohort	Age groups (18-43yrs). Male 98% vs. 98%	48 pts **Lyo** Plasma and pRBC (1:1) vs. 48 matched standard care (500 mL of Hartmann Solution)	**NA**	HR > 130 bpm, lack of radial pulse, and/or SBP < 90 mm Hg	Lyoplas use had no improvement in casualty’s outcome, LOS or mortality at discharge (6.2% vs. 8.5%, *p* = 0.17)	Hemoglobin (*p* = 0.03) and platelets (*p* = 0.04) were lower in the Lyoplas group.
Mitra 2023 [28] June-Oct 2022	RCT, Multicenter, Australia (**PILOT**)	48 vs. 34 yrs, Male 78% vs. 64%	9 patients (**Lyo** Plasma; Lyoplas) vs. 11 patients standard care	**Air**	Suspected hemorrhage and hypovolemia. A target SBP ≥ 70 mm Hg or, if there is concurrent severe TBI, a target SBP ≥ 120 mm Hg.	Mortality was not significantly lower in the **FDP** group at 24 h (RR 0.24**)** and at hospital discharge (RR 0.73). No serious adverse events related to the intervention	The median number of units of PH RBCs was 3 (2–4) and equal in both arms. No tranexamic acid was given. The median time from randomization to hospital arrival was 92.5 min.
Kim 2012 [18] Feb 2009 -May 2011	**Retrospective**, **Single**-center, USA	41 vs. 54 yrs. Male 60% vs. 89%	50 controls vs. **9** thawed plasma and pRBC (2.1U:2.5U) in PH. Control group received 1 unit of RBC PH	**Air**	≥ 2 of: (1) single reading of SBP ≤ 90 mm Hg, (2) single reading of HR ≥ 120, (3) penetrating mechanism, (4) POC lactate of ≥ 5.0 mg/dL or (5) POC INR of ≥ 1.5	6 h mortality, transfusion-related complications and hospital and ICU stays were similar, The study group had significantly **higher** overall (56% vs. 18%) and 24-hour (44% vs. 10%) mortality rates. Transfusion-related complications and hospital and ICU were similar	The ‘‘geographic plasma deficit’’ is magnified by mean transport time being 40 min
Crombie 2022 [13] Nov 2016 - Jan 2021 RePHILL	**RCT**, Multicenter, UK	38 vs. 39 yrs, Males 82% in both groups	209 **Lyo** Plasma and pRBC (1:1) vs. 223 NLS (up to 4 bags)	**Air** 38% & **Ground** 62%	Adults (age ≥ 16 yrs) with injury and SBP < 90 mm Hg or absence of palpable radial pulse)	pRBC–Lyoplas **did not** improve episode mortality or lactate clearance when compared with 0·9% sodium chloride for patients with trauma-related hemorrhagic shock	Before randomization, participants received an average of 430 mL crystalloids and tranexamic acid. Median ISS 36. Median EMS time 83 min.

The first randomized study of prehospital use of lyophilized plasma to assess its effect on INR; * * leukocyte-depleted; 80% of platelets in whole blood donations were removed, and the remaining component contained red cells and plasma in one bag. Ω: adjusted for injury, age, HR, and SP, PH: prehospital; PHP = prehospital plasma; NLS: normal saline. aHR: adjusted hazard ratio; PH: prehospital transfusion; IH: in-hospital transfusion; SBP: systolic blood pressure, FAST: Focused Assessment with Sonography in Trauma; MOT: mode of transportation; HR: heart rate; MOF: multiorgan failure; pRBCs: packed red blood cells, RCT: randomized clinical trial; ISS: injury severity score; PHT: blood product transfusion prehospital; ITT: intension to treat; Lyo lyophilized; SI: shock index; FDP: freeze-dried plasma; RR: relative risk; LOS: length of stay

**Table 2:** Studies included in the systematic review but not in the meta-analysis *n* = 3

Author/Year/duration	Study design/country	Age and sex per group	*N* of patients per group	MOT	Indication	Outcomes	Comment
Oakeshott et al. [31] 2019 April 2013 - December 2016.	Retrospective, single-center, UK	mean age was 46 yrs (4–90 yrs) and 73% Male (no data for the pRBC gp)	85 (42 pRBC and FDP, 40 FDP only and 3 pRBC only) vs. 79 pRBC gp (one year prior)	**Air**	SBP < 80 mmHg or absence of a radial pulse	There was an 18% reduction in pRBC amount for the Plasma group compared with the pRBC-only group. No reported adverse reactions	Earlier transfusion of FDP and pRBC was feasible, the median time to arrive at the hospital was 111 min (IQR: 95–138 min). No mortality data
*Henrisken 2016 [2] Oct 2012 - Nov 2013	**Prospective**, **Single**-center, USA	≥ 16 yrs (median 34) & 83% male	Compare 75 PH (prehospial RBCs and thawed plasma) vs. 182 IH (RBCs, plasma and platelet within 6 h after arrival).	**Air** and **Ground**	**PH** was associated with more penetrating injuries, more positive FAST, lower SBP, lower hemoglobin, lower platelet count, lower pH, and lower rTEG	**PHP** is more beneficial than prehospital **RBCs** for improving hemostasis	72/75 received PHP. Only 13 of these 72 have not received PRBC. **In the PH** group, nearly half received both RBCs and plasma. Despite more severe injury and worse clinical presentation, the **PH** group had early and late mortality similar to the **IH** group
Shackelford et al. 2017 [15] April 2012, and August, 2015	Retrospective cohort study of USA military combat	Median age, 25 yrs Male 98% (total population)	55 **PHT** (38 only RBCs; 7 thawed plasma and 10 received one unit from each) vs. 345 matched nonrecipients	**Air**	≥ 1 traumatic limb amputation with at least one located above the knee or elbow, or shock defined as SBP < 90 mm Hg or HR > 120)	24-hrs (3 deaths among 54 recipients vs. 67 deaths among 332 matched nonrecipients): aHR for mortality associated with prehospital transfusion was 0.26, *P* = 0.02) and for 30-day mortality aHR 0.39, *P* = 0.03) (6 vs. 76 deaths, respectively).	Military trauma system. On sensitivity analysis, despite removing > 50% of prehospital deaths, 24-hour mortality was still significantly decreased for recipients of transfusions started within about 36 min after injury

PH: prehospital; PHP = prehospital plasma; NLS: normal saline. aHR: adjusted hazard ratio; PH: prehospital transfusion; IH: in-hospital transfusion; SBP: systolic blood pressure, FAST: Focused Assessment with Sonography in Trauma; MOT: mode of transportation; HR: heart rate; pRBCs: packed red blood cells, PHT: blood product transfusion prehospital; FDP: freeze-dried plasma; gp: group; IQR: interquartile range

Three studies assessed the combined effect of plasma and pRBC, rather than the independent effect of plasma alone [13, 17, 30]. In Holcomb et al. [17], among patients receiving prehospital transfusion (PHT), 24% received only plasma, 7% received only RBCs, and 69% received both. These three studies were included in the primary meta-analysis. Still, they were later excluded from the sensitivity analysis to assess their impact on the overall results because the intervention consisted of plasma and/or packed red blood cells (pRBC). In contrast, studies by Oakshott et al. and Shackelford et al., in which only a portion of the intervention group received plasma, were excluded from all quantitative analyses. Holcomb et al. (PROHS) [17] analyzed three population subsets; however, only the propensity-matched analysis was utilized in our analysis. Regarding the type of plasma used, five studies used thawed plasma [2, 5, 15, 17–19], five utilized FDP [13, 27, 28, 30, 31], and one used both [29]. Tucker et al. [29] conducted the largest study, involving 686 patients in the intervention group, followed by Sperry et al. (PAMPer trial) [5], which included 230 patients in the intervention group.

### Quality assessment findings (NOS and ROB2)

The quality of 5 observational studies [2, 15, 17, 29, 30] was good, reflecting methodology with an adequate selection of cohorts, robust comparators, and reliable outcome assessment. However, two studies were of moderate quality, primarily due to a lack of adjustment for confounding factors, which introduced potential selection bias [18, 31] (Suppl Table 2). The RCTs had some concerns, especially in domain 2 (risk of bias due to deviations from the intended interventions), but were generally at low risk for all the other domains. The small sample size in the plasma group, particularly in Mitra et al. [28], may contribute to variability in the results. (Suppl Table 3).

### Outcomes

Eight studies assessed mortality as an outcome; two focused on laboratory hemostatic variables, such as INR or rTEG [2, 27], and one evaluated the impact on total pRBC transfusion. Short-term mortality was considered either 3-hour mortality in one study (OR 0.74, *P* = 0.60) [17] or 6-hour mortality in two studies, with no significant difference noted [18, 27]. Sperry et al. [5] observed improved 30-day mortality in the plasma group, whereas Tucker et al. [29] reported reduced 24-hour mortality in the PHP group; however, this benefit did not extend to the 30-day outcome. Kim et al. [18] also demonstrated improvements in 24-hour mortality; however, their study included only nine patients in the intervention group. Mitra et al. and Shlaifer et al. reported no significant mortality difference at discharge.

Shackelford et al. [15] reported a significant improvement in the intervention group for 24-hour and 30-day mortality rates. In this study, the intervention was either pRBC, plasma, or a combination; only 17 of 55 patients received plasma. Oakeshott et al. [31] found that the use of prehospital FDP reduced the use of prehospital packed red blood cells (pRBC). The other studies did not show significant improvement in mortality or hemostatic parameters with PHP transfusion. All studies highlighted the practicality and feasibility of PHP. The volume of PHP transfused within 24 h varied across studies, with two studies [17, 18] showing a significant increase in PHP usage as compared to the control group. Similarly, 24-hour pRBC transfusion volumes varied across studies. Sperry et al. [5] reported a significant decrease in pRBC use in the PHP group vs. the no-PHP group, whereas Holcomb et al. [17] observed a significant increase in pRBC administration. Other studies found no significant difference, though variability between the groups was evident.

#### Twenty-four-hour Mortality

**Fig. 2:**
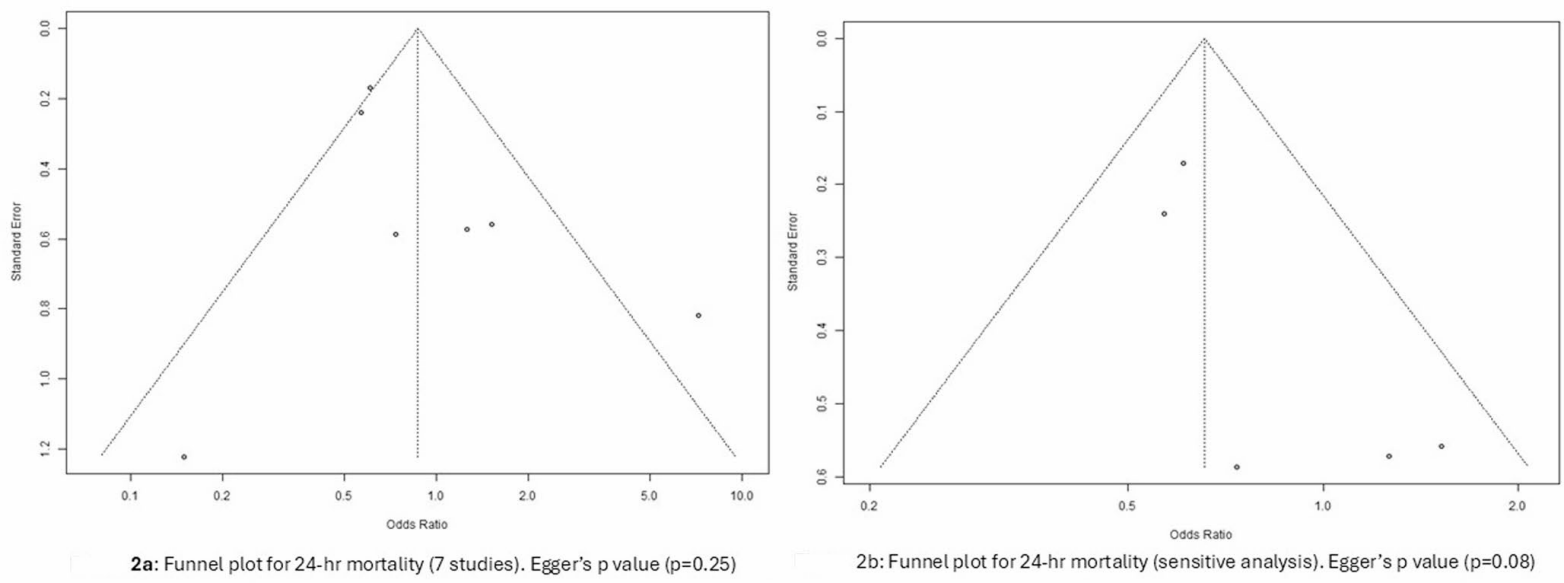
Funnel plot for 24-hr mortality (7 studies). Egger’s p value (p=0.25). Funnel plot for 24-hr mortality (sensitive analysis). Egger’s p value (p=0.08)

Seven studies evaluated 24-hour mortality, including 2,170 patients. Despite the pooled effect of PHP use (RCT and non-RCT studies) showing lower 24-hour mortality compared to controls, the difference was not statistically significant [RR 0.92 (95% CI: 0.62–1.38)] (Fig. 2a). The studies showed moderate heterogeneity (I^2^ = 58.8%). Studies by Kim et al. [18] and Mitra et al. [28] showed high variances due to smaller sample sizes; therefore, both were excluded from calculating the effect size. In addition, the study by Holcomb et al. [17] was excluded from sensitivity analysis because the intervention consisted of plasma and/or RBC.

Figure 2b illustrates the forest plot and sensitivity analysis based on four studies. This pooled analysis favored PHP transfusion over the control group [RR = 0.76; 95% CI, 0.61–0.94] in reducing 24-hour mortality. The two small-sample-size studies [18, 28] had weights of 9% and 4% in the random effect model. They largely contributed to heterogeneity (>50% I-squared value). After removing these three studies, the heterogeneity dropped considerably (I^2^ = 14%). For the overall analysis, including these three studies, the fixed-effects model showed a significant difference; however, due to heterogeneity, we opted for the random-effects model.

#### Late mortality

Fig. 2c illustrates late mortality from six studies (I^2^ = 0.0%). The pooled effect indicated a reduction in mortality with PHP compared to the control group. However, the finding was not statistically significant [RR = 0.89, (95% CI: 0.80–1.00)]. Figure 2d shows the sensitivity analyses by removing Crombie et al. and Holcomb et al., as they had a combined intervention, yielding similar results [RR = 0.87, (95% CI: 0.71–1.08)].

#### Subgroup analyses

**Fig. 3:**
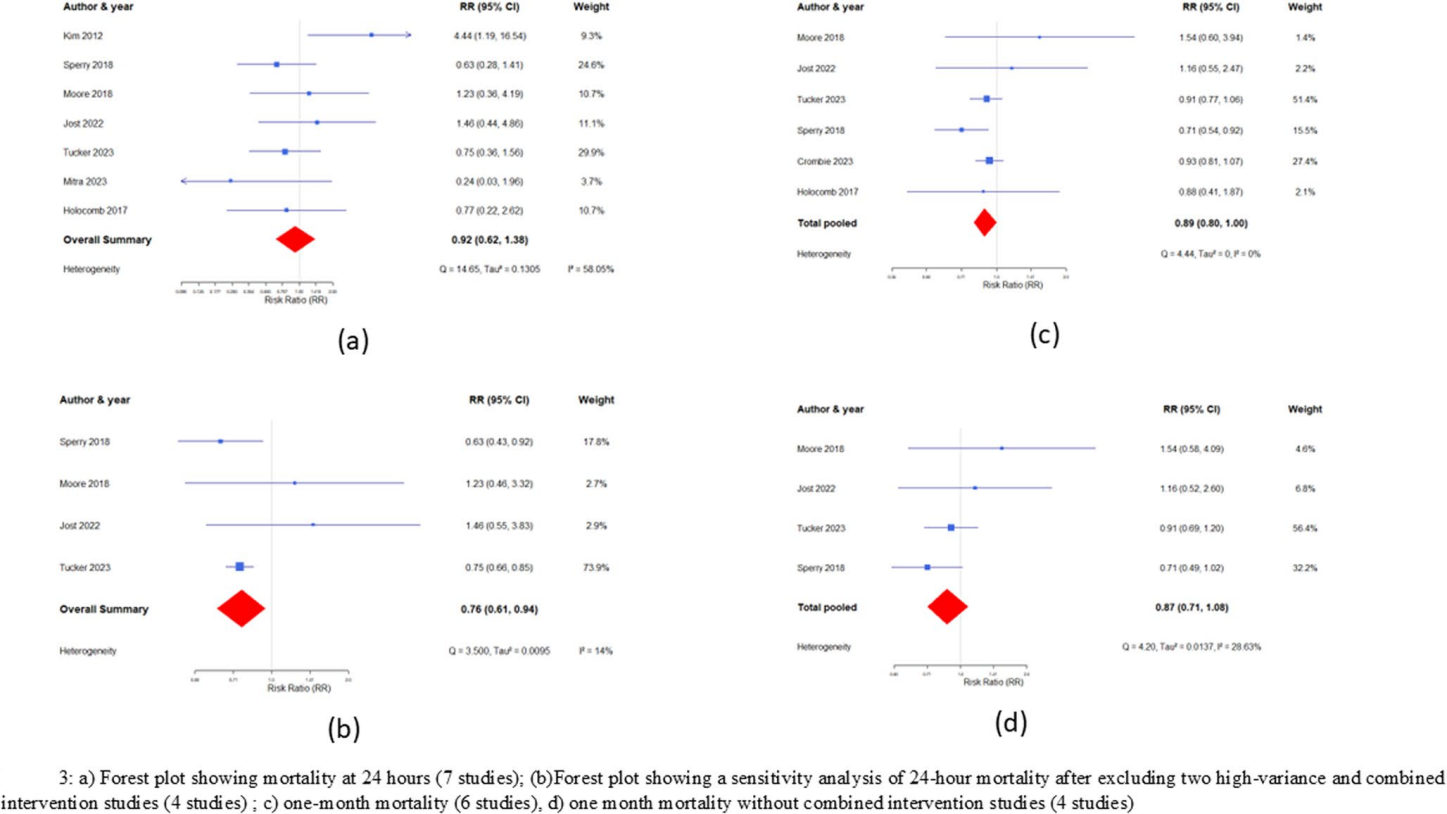
(**a**) Forest plot showing early mortality at 24 hours (7 studies); (**b**)Forest plot showing a sensitivity analysis of 24-hour mortality after excluding two high-variance and combined intervention studies (4 studies);(**c**) Late mortality at 28 or 30 days (6 studies), (**d**) Late mortality without combined intervention studies (4 studies)

A subgroup analysis (Fig. 3) included four homogeneous studies based on study type (RCT (COMBAT, PAMPer, and PREHO-PLYO) vs. non-RCT (Tucker et al.), mode of transportation (air vs. ground), and type of plasma used (thawed vs. lyophilized). The results showed no significant difference in RCTs [RR = 0.89, 95% CI: 0.50–1.57], whereas non-RCTs (Tucker et al.) indicated a significant effect [RR = 0.75, 95% CI: 0.66–0.85] for 24-hour mortality. Thawed plasma alone showed a decreased risk [RR = 0.75, 95% CI: 0.42–1.33], although this was not statistically significant. In terms of transportation, air transport showed a significant effect [RR = 0.72, 95% CI: 0.61–0.86], whereas ground transport did not [RR = 1.34, 95% CI: 0.67–2.70]. Notably, 74% of the pooled effect of the four homogeneous studies was driven by a single study by Tucker et al. [29], which was a non-randomized controlled trial (RCT) using lyophilized and thawed plasma in air transport.

#### Secondary outcomes

The pooled analysis demonstrated no statistically significant difference between the PHP transfusion and the control group for hematological parameters.

The total 24-hour volume of plasma units transfused was higher than that of the control group [MD: 0.46 (95% CI: −0.47–1.38)].

Despite the total 24-hour volume of RBC units [MD: − 0.57 (95% CI: − 1.78 − 0.63)] and vasopressor use being lower in the plasma group [RR: 0.91 (95% CI: 0.78–1.07)], the difference did not reach statistical significance.

**Fig. 4:**
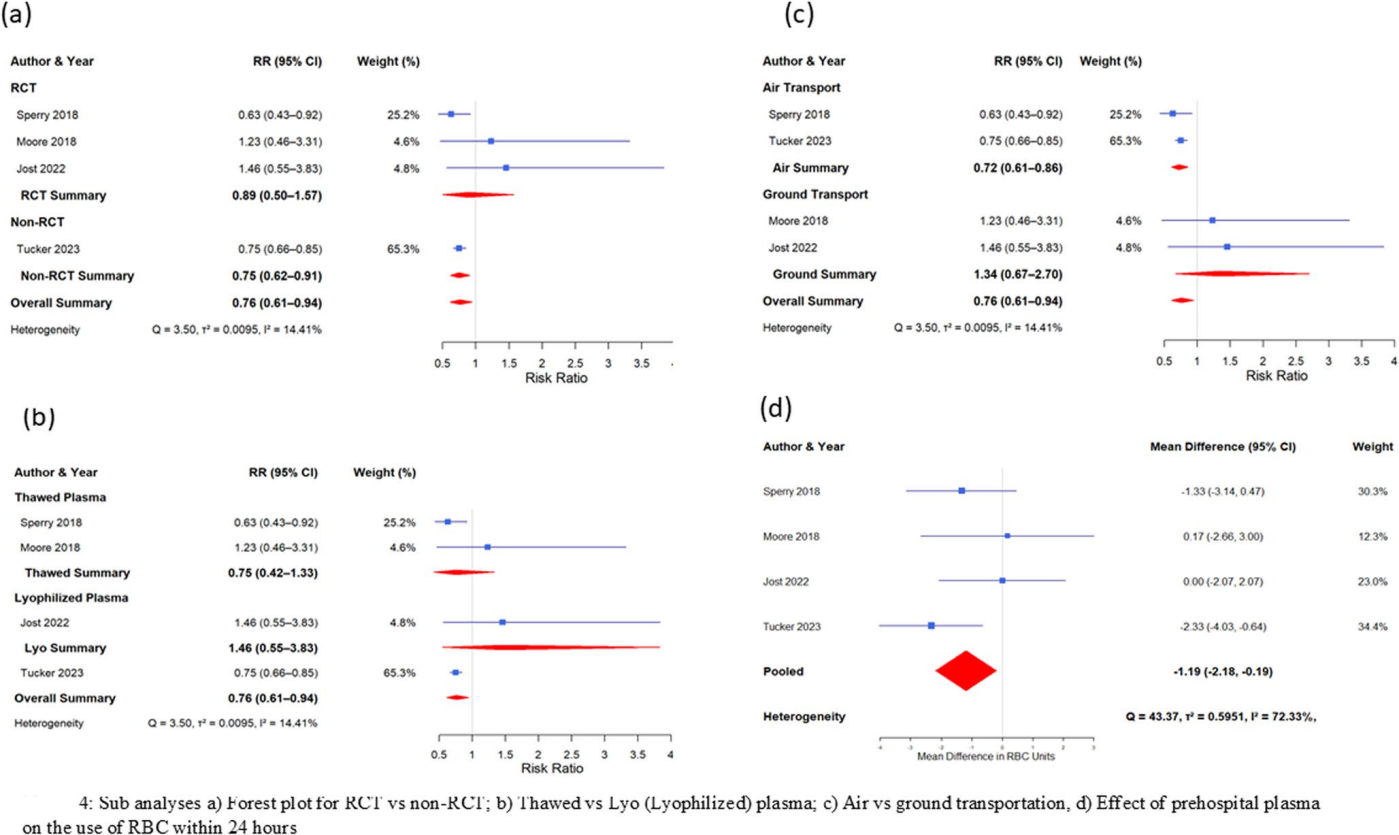
Sub-analyses **a**) Forest plot for RCT vs non-RCT; **b**) Thawed vs Lyo (Lyophilized) plasma; **c**) Air vs ground transportation; **d**) Effect of prehospital plasma on the use of RBC within 24 hours

After removing the combined intervention study, Crombie et al. showed a significant decrease in RBC use [MS: −1.19 (95% CI: −2.18−0.19)] (Fig. 4).

There was no significant difference between PHP transfusion and control groups in the incidence of MOF [RR: 1.08 (95% CI: 0.96–1.20)], adverse events [RR: 1.09 (95% CI: 0.63–1.87)], ALI [RR: 0.99 (95% CI: 0.77–1.28)], transfusion reactions [RR: 1.64 (95% CI: 0.15–18.07)] and sepsis [RR: 0.77 (95% CI: 0.39–1.52)](Supplementary figure).

Removing the combined intervention studies did not substantially alter the results, and the findings remained non-significant.

The GRADE assessment revealed low-certainty evidence for 24-hour mortality (RR 0.76, 95% CI 0.61 − 0.94), primarily downgraded due to the risk of bias and indirectness. Evidence for late mortality (RR 0.87, 95% CI 0.71 − 1.08) was deemed very low certainty due to risk of bias, indirectness, and imprecision. Similarly, the 24-hour pRBC transfusion requirement (MD − 1.19, 95% CI − 2.18 to − 0.19 units) was supported by very low certainty evidence, downgraded for risk of bias, inconsistency, and indirectness (Supplementary Table 4). Most studies were funded by government or national health bodies, with no role reported for funders in study conduct. In a few cases, such as Shlaifer et al. and Tucker et al., the funders’ roles were not clearly stated (Supplementary Table 5).

#### Publication bias

Funnel plots, available in the appendix, were used to assess publication bias. Egger’s p-values were also provided, none of which were statistically significant (Fig. 4).

#### Umbrella review

Umbrella review was not conducted due to the heterogeneity and inconsistent inclusion criteria and outcomes. However, the seven available SRs were summarized for the readers to clarify this debatable point and show the main differences between the SRs (Table 3).

**Table 3:** Characteristics of published systematic reviews (SR) related to prehospital plasma transfusion

Authors	Number of studies	Civilian/military	Quality	Comparative population	Study design	Comment
Rijnhout et al. 2019 [20]	49 studies in SR and 9 studies in meta-analysis	Both	Two RCTs had a high risk of performance bias. Among the cohort studies, six had a critical risk of bias, while one had a serious risk.	Standard care	2 RCT/7 retro (RMC)	PHBT with simultaneous use of both pRBCs and plasma resulted in a significant reduction in the odds for long-term mortality. PHBT is safe.
Jackson et al. 2021 [21]	4 primary studies and 10 secondary studies	Civilian	No quality check done. The inclusion of secondary studies and mixed study designs may have introduced bias.	Standard care	1 retro/1 prosp/2 RCTs (primary)	PHP may provide a mortality benefit, particularly in patients with blunt injuries, moderate transfusion needs, TBI, transport time > 20 min, or specific cytokine profiles.
Mok et al. 2021 [24]	12 human and 15 Animal studies	Both	Evidence was of low quality for both mortality and exposure to ABPs. Included in-hospital/out-of-hospital (received FDP within 24 h. The prehospital settings were not explicit. Heterogeneous populations.	Frozen plasma, factor concentrates, or goal-directed therapy (laboratory-guided, TEG, or ROTEM).	8 retro/3 prosp/1RCT (Human)	Human data assessing FDP use in trauma report no difference in mortality and transfusion of ABPs in patients receiving FDP compared with FP.
Sheffield et al. 2024 [4]	3 RCTs	Civilian	NA	Normal saline/PRBC alone	3 RCT	No clear mortality or INR benefit, feasibility noted for long transport.
AlJoaib et 2024 [22]	3 RCT	Civilian	High ‘performance and detection’ bias	Normal saline	3 RCT	There is no distinction between PHP and normal saline concerning mortality at 24 and 28 days or the need for vasopressors within 24 h. Plasma administration did not appear to influence rates of acute lung injury or multiorgan failure.
Abuelazm et al. 2024 [23]	3 RCTs	Civilian	Some concern due to deviation from the intended intervention	Standard-care resuscitation using crystalloid solution infusion	3 RCT	Plasma infusion in trauma patients at risk of hemorrhagic shock does not significantly affect mortality or the incidence of multiple organ failure. However, it may lead to reduced packed red blood cell transfusions and increased INR at hospital arrival.
Coccolini et al. 2019 [25]	2 RCTs	Civilian	Reported both papers as High quality	Standard care	2 RCT	Prehospital plasma infusion seems to reduce 24-hour mortality in hemorrhagic shock patients. It does not seem to influence 1-month mortality, acute lung injury, and multiorgan failure rates.

RMC = retrospective matched cohort, PHBT: prehospital blood transfusion, retro: retrospective, prosp: prospective, ABPs: Allogenic blood products FDP: freeze dried plasm

## Discussion

The present SR and meta-analysis on the efficacy and safety of PHP transfusion in trauma patients analyzed the mortality, laboratory parameters, need for blood transfusions, and complications. The key findings highlighted that the pooled outcome after sensitivity analysis favored PHP transfusion over the control group receiving isotonic saline solution, RBC, or other crystalloids in reducing 24-hour mortality. However, PHP alone or combined with pRBC did not significantly impact late mortality or corrected hematological parameters such as INR or rTEG. However, the certainty of evidence for 24-hour mortality was rated as low according to GRADE. For 30-day mortality and the use of RBC within 24 h, the rates were very low, substantially limiting confidence in these findings and reinforcing the need for larger, well-designed randomized trials. The total 24-hour volume of plasma units administered was higher in patients receiving PHP. Although not statistically significant, the PHP group showed a reduction in the total 24-hour volume of RBC units and vasopressors compared to the control group. Similarly, the incidence of MOF, adverse events, transfusion reactions, and sepsis did not differ significantly between the PHP transfusion and control group. The mortality benefit after PHP was shown in non-RCT (one study), air transportation (two studies), lyophilized plasma (one study), and short-term outcomes (four studies).

The heterogeneity across studies should be noted. Differences in study design partly explain the inconsistent results: combined RCTs showed no survival benefit, while observational studies suggested early mortality reduction, likely reflecting residual confounding. Patient populations also varied, with some studies including blunt trauma and others more penetrating injuries, as well as different injury severities and prehospital shock indices. The type of plasma used may have influenced the outcomes of the patients. Finally, the transport mode appeared to be important as the benefit was more evident in air-transported patients. The findings from subgroup analyses should be interpreted with caution. These analyses were limited by small sample sizes within each subgroup and a lack of adjustment for potential confounders. For example, the apparent benefit seen in non-RCTs and air-transported patients may reflect differences in injury severity, transport time, or concurrent treatments rather than an actual effect of plasma. Similarly, differences by plasma type may be influenced by study setting and logistics rather than inherent biological efficacy. As such, the subgroup results are hypothesis-generating and not conclusive.

A recent multicenter study [32] showed that patients receiving prehospital blood product transfusion (PHBPT) were more severely injured with a higher scene shock index (SI) than those not receiving PHBPT. In the ED, patients receiving PHBPT showed significantly lower SI and better survival with lower Trauma Injury Severity Score (TRISS); however, 30-day mortality was similar in the two groups. We did not include this study in our analysis as the number of patients who received plasma was not explicitly described in the results and outcomes. Prehospital blood components have significantly increased the efficacy of the early DCR approach in trauma patients [33–36]. However, this requires specific storage and handling capabilities, such as refrigerated spaces, strict manipulation procedures, and administrative oversight. Such challenges are compounded by the need to constantly renovate the prehospital blood stocks, considering their short lives and the risk of wasting unused products [17]. Other transfusion-related challenges include the demand for universal blood products, which are often the rarest, and compliance with strict and complex regulatory principles [13]. Conversely, an earlier SR reported that, although pre-hospital blood transfusion is a plausible therapeutic approach, the supporting evidence of benefit was of poor quality and did not show outcome improvements [37, 38].

Comparatively, less attention has been given to the use of plasma alone for prehospital resuscitation [39]. Similar to our findings, a recent meta-analysis reported a significant reduction in 24-hour mortality in patients receiving PHP transfusion compared to controls [25]. In contrast, two recent meta-analyses [22, 23] observed no survival benefit after 24 h with the administration of PHP. Studies and SRs did not demonstrate consistency in the inclusion criteria, study settings, outcome measures such as age, sex, systolic blood pressure (SBP), emergency medical service (EMS) time, mode of injury and transportation, patients injury severity score (ISS), use of tranexamic acid, evidence of coagulopathy, whether plasma was given before or after pRBCs, types of plasma and standards of care, and study power and quality assessment. These factors should be considered for the generalizability of the studies and SR results. Conflicting arguments persist, including that pRBC transfusion could reduce the patient’s relative residual plasma if transfused before plasma (hemoconcentration). On the other hand, plasma transfusion could decrease the hematocrit (hemodilution) and oxygen delivery if given first [4]. In the RePHILL study, PRBC was administered before LyoPlas, whereas in PAMPer, plasma was administered first. RePHILL patients received an average of 430 mL of crystalloid before randomization, and 90% also received tranexamic acid. PAMPer patients were older, had higher rates of blunt injury and lower ISS scores, and were subjected to longer transit times than those in the COMBAT study. The longer the time, the more benefit from PHP. The ISS values were also higher in RePHILL than in the PAMPer study. The use of tranexamic acid in combination with FFP or FDP appears to improve patient outcomes in hemorrhagic shock.

Moreover, our analysis demonstrated that plasma alone or combined with pRBC did not significantly impact in-hospital and/or late mortality in trauma patients, which agrees with earlier meta-analyses [22, 23]. Notably, the findings of the meta-analyses were based on only 3 RCTs. However, in the PAMPer trial [5], the PHP group experienced a significant decrease in 30-day mortality (23% vs. 33%). However, this survival benefit did not persist in prespecified subgroups except with prolonged transport time and blunt traumatic injuries. The COMBAT trial [19] was conducted in an urban setting with short transport times (< 30 min); however, it was discontinued early due to treatment futility, with no significant difference in 28-day mortality. In the PREHO-PLYO [27], lyophilized PHP was compared to crystalloids. It resulted in no significant difference in primary (prevention of TIC defined by the admission INR) and secondary outcomes (massive transfusion, 30-day survival, and safety). It is crucial to recognize that, although the hemodynamic effects of PHP may be clinically beneficial, these effects did not translate into measurable outcomes, such as lower mortality.

Furthermore, even if PHP could correct or prevent TIC, it may not result in measurable clinical improvements, such as better survival. Conversely, the non-measured effects of administering PHP on immunological, hemodynamic, or physiological parameters can theoretically be extrapolated to contribute to better outcomes [40]. Notably, it is very challenging to significantly decrease mortality in the complex population of bleeding trauma patients, where the interaction of innumerable factors determines death. This requires ensuring that the groups are matched and comparable, which is a challenge in the prehospital and even early hospital phases, necessitating strict protocols that account for all pragmatic and ethical considerations. PHP transfusion aims to provide volume and early replacement, or pre-emptive loading, of coagulation factors in an actively bleeding patient [2]. A meta-analysis by Mok et al. compared the use of FFP and FDP in animal and human studies (within 24 h of assessment) [24]. Findings from animal studies indicated that FDP and FFP produced comparable coagulation and anti-inflammatory profiles. Only two human studies were analyzed, revealing no significant differences in 30-day mortality [24].

Another important outcome studied in our analysis was the need for RBC transfusions. The findings revealed a non-significant decrease in the total 24-hour volume of pRBC units and vasopressors in the PHP group. These results align with an earlier meta-analysis [23]. Concerning laboratory parameters, Henriksen et al. [2] reported improvements in rTEG maximum amplitude in the plasma group, which arguably indicates an improved coagulation status. PHP had no measurable effect on ordinary laboratory tests such as INR.

The safety outcomes analysis demonstrated that plasma did not increase the risk of MOF, ALI, transfusion reactions, or sepsis. Our findings are similar to those of Al Joaib et al. [22], who reported that PHP administration had no significant effect on MOF and ALI. A meta-analysis by Abuelazm et al. [23] also found no significant differences regarding the rates of MOF and sepsis. However, these safety findings should be interpreted with caution. Adverse events may have been under-reported in the included studies, and none were adequately powered to detect rare but clinically meaningful complications such as transfusion-related acute lung injury (TRALI) or allergic reactions. Moreover, safety was not the primary outcome in most trials, further limiting confidence in the evidence. Thus, while the available data appear reassuring, the accurate safety profile of PHP remains uncertain. Despite the reassurances from earlier SRs, the safety of plasma transfusion remains a concern until large RCTs address it as a primary outcome in prehospital settings. The prehospital FDP host inflammatory response, safety, and efficacy remain unknown [24].

## Strengths and limitations

This SR provides an updated and comprehensive synthesis of the evidence on PHP administration in trauma patients, incorporating data from nine studies. In comparison, two recently published meta-analyses on the same topic [22, 23] were limited to only three overlapping RCTs. Another key strength of the present analysis is its rigorous methodology. We conducted a meticulous literature search to ensure comprehensive coverage of relevant studies. By applying predefined inclusion and exclusion criteria, we ensured that only high-quality and relevant studies were incorporated, reducing the risk of selection bias.

Additionally, the quality of the included studies was systematically assessed, further strengthening the credibility and robustness of our findings. Although we reviewed seven SRs, we were unable to conduct an umbrella review due to the heterogeneity and inconsistent inclusion criteria and outcomes. The lack of information on the causes of prehospital deaths could influence the real occurrence of TIC and transfusion reactions and their effect on the outcomes.

On the other hand, certain limitations of this meta-analysis need attention. First, some of the included studies were primarily designed to assess feasibility rather than efficacy or safety, which may introduce biases in outcome reporting. Additionally, some of the studies were non-randomized, retrospective, or prospective observational studies, which inherently have a higher risk of confounding factors than RCTs. The heterogeneity in study design and population characteristics may also limit the ability to draw definitive conclusions for trauma patients. As this meta-analysis was conducted using study-level data, confounding effects related to factors such as dose, volume, timing, and injury severity could not be adjusted since the original studies did not consistently report or control for these variables. Second, the variation observed in the findings of Kim et al. [18] and Mitra et al. [28] compared to other studies could be attributed to the small sample size in these studies, particularly in the PHP group. This limitation underscores the potential for bias and reduces the generalizability of these findings to broader patient populations. Moreover, the inclusion of multiple comparator groups in the Tucker et al. [29] study, with differing treatment modalities, complicates direct comparisons and may affect the robustness of the pooled estimates. Despite a comprehensive search, publication bias also cannot be excluded, as small negative studies and adverse events may be underreported.

One significant limitation is the reporting of mortality at varying time points (e.g., 24-hour, 28-day, and in-hospital mortality/follow-up), as these outcomes were not standardized across the included studies. For example, some studies report 24-hour mortality, while others report 3- or 6-hour mortality. The inconsistency in outcome definitions across the included studies introduces heterogeneity and limits the comparability of results. To address this issue, we recommend adopting standardized mortality time points as outlined in the Core Outcome Set for Trauma Research (COS-TREND) and Utstein-style trauma reporting templates, which advocate for defining mortality outcomes at 24 h, 7 days, and 30 days post-injury [41, 42]. Incorporating these consensus-based endpoints in future trials would strengthen the interpretability and synthesis of evidence. This would enhance comparability for future meta-analyses. Additionally, subgroup analyses based on outcome definitions should be conducted to assess the consistency of findings across studies. Further studies should focus on standardized endpoints and enhance the reporting of adverse events.

## Conclusions

Using sensitivity analysis, the present meta-analysis revealed that PHP transfusion was associated with a reduction in 24-hour mortality in trauma patients. However, this finding is based on low-certainty evidence. No benefit was observed for longer-term mortality. Overall analysis and seven prior SRs showed no consistent survival benefit. PHP may reduce the need for pRBC transfusion within the first 24 h, but does not significantly decrease vasopressor use. PHP administration is safe, but further studies are warranted to adopt this outcome. The present review of the SRs and meta-analysis provides a foundation for future studies to address the limitations and confounding factors existing in the current literature, which may lead to the development of optimized transfusion protocols in prehospital emergency care settings.

## Supplementary Information

Below is the link to the electronic supplementary material.

Supplementary Material 1 (DOCX 26.7 KB)

Supplementary Material 2 (DOCX 122 KB)

## Data Availability

No datasets were generated or analysed during the current study.

## Declarations

### Presentation

This study was presented partly at the ECTES: 24th European Congress of Trauma and Emergency Surgery, 13-15 April 2025, Aachen, Germany.

### Registration

The protocol is registered and available on PROSPERO ID: CRD42024599358.

## Abbreviations

SRs: Systematic reviews
PHP: prehospital hospital plasma
FDP: fresh dried plasma
FFP: fresh frozen plasma
TIC: trauma-induced coagulopathy
pRBC: packed red blood cells

## References

[1] Evans JA, van Wessem KJ, McDougall D, Lee KA, Lyons T, Balogh ZJ. Epidemiology of traumatic deaths: comprehensive population-based assessment. World J Surg. 2010;34(1):158–63. 10.1007/s00268-009-0266-1. 19882185 10.1007/s00268-009-0266-1

[2] Henriksen HH, Rahbar E, Baer LA, Holcomb JB, Cotton BA, Steinmetz J, et al. Prehospital transfusion of plasma in hemorrhaging trauma patients independently improves hemostatic competence and acidosis. Scand J Trauma Resusc Emerg Med. 2016;9(1):145. 10.1186/s13049-016-0327-z. 10.1186/s13049-016-0327-z PMC5148857 27938373

[3] Whittaker B, Christiaans SC, Altice JL, Chen MK, Bartolucci AA, Morgan CJ, Kerby JD, Pittet JF. Early coagulopathy is an independent predictor of mortality in children after severe trauma. Shock. 2013;39(5):421–6. 10.1097/SHK.0b013e31828e08cb. 23591559 10.1097/SHK.0b013e31828e08cb PMC3689548

[4] Sheffield WP, Singh K, Beckett A, Devine DV. Prehospital freeze-dried plasma in trauma: a critical review. Transfus Med Rev. 2024;38(1):150807. 10.1016/j.tmrv.2023.150807. 38114340 10.1016/j.tmrv.2023.150807

[5] Sperry JL, Guyette FX, Brown JB, Yazer MH, Triulzi DJ, Early-Young BJ, Adams PW, Daley BJ, Miller RS, Harbrecht BG, Claridge JA, Phelan HA, Witham WR, Putnam AT, Duane TM, Alarcon LH, Callaway CW, Zuckerbraun BS, Neal MD, Rosengart MR, Forsythe RM, Billiar TR, Yealy DM, Peitzman AB, Zenati MS. PAMPer study Group. Prehospital plasma during air medical transport in trauma patients at risk for hemorrhagic shock. N Engl J Med. 2018;379(4):315–26. 30044935 10.1056/NEJMoa1802345

[6] Barelli S, Alberio L. The role of plasma transfusion in massive bleeding: protecting the endothelial glycocalyx? Front Med Lausanne. 2018;5:91. 29721496 10.3389/fmed.2018.00091 PMC5915488

[7] Zur M, Glassberg E, Gorenbein P, Epstein E, Eisenkraft A, Misgav M, Avramovich E. Freeze-dried plasma stability under prehospital field conditions. Transfusion. 2019;59(11):3485–90. 31568580 10.1111/trf.15533

[8] Garrigue D, Godier A, Glacet A, Labreuche J, Kipnis E, Paris C, Duhamel A, Resch E, Bauters A, Machuron F, Renom P, Goldstein P, Tavernier B, Sailliol A, Susen S. French lyophilized plasma versus fresh frozen plasma for the initial management of trauma-induced coagulopathy: a randomized open-label trial. J Thromb Haemost. 2018;16(3):481–9. 29274254 10.1111/jth.13929

[9] Glassberg E, Nadler R, Gendler S, Abramovich A, Spinella PC, Gerhardt RT, et al. Freeze-dried plasma at the point of injury: from concept to doctrine. Shock. 2013;40(6):444–50. 10.1097/SHK.0000000000000047. 24089000 10.1097/SHK.0000000000000047

[10] Holcomb JB, Donathan DP, Cotton BA, Del Junco DJ, Brown G, Wenckstern TV, et al. Prehospital transfusion of plasma and red blood cells in trauma patients. Prehosp Emerg Care. 2015;19(1):1–9. 10.3109/10903127.2014.923077. 24932734 10.3109/10903127.2014.923077

[11] Cardenas JC, Holcomb JB. Time to plasma transfusion: a patient centered approach and modifiable risk factor. Transfusion. 2017;57(4):869–73. 10.1111/trf.14019. 28394421 10.1111/trf.14019

[12] Nielsen JS, Watson J. Damage control resuscitation and surgery in a forward combat setting. Curr Trauma Rep. 2016;2:165–72. 10.1007/s40719-016-0049-8.

[13] Crombie N, Doughty HA, Bishop JRB, Desai A, Dixon EF, Hancox JM, et al. Resuscitation with blood products in patients with trauma-related haemorrhagic shock receiving prehospital care (RePHILL): a multicentre, open-label, randomised, controlled, phase 3 trial. Lancet Haematol. 2022;9(4):e250. 10.1016/S2352-3026(22)00040-0. 35271808 10.1016/S2352-3026(22)00040-0 PMC8960285

[14] Radwan ZA, Bai Y, Matijevic N, del Junco DJ, McCarthy JJ, Wade CE, Holcomb JB, Cotton BA. An emergency department thawed plasma protocol for severely injured patients. JAMA Surg. 2013;148(2):170–5. 10.1001/jamasurgery.2013.414. 23426594 10.1001/jamasurgery.2013.414 PMC3800103

[15] Shackelford SA, Del Junco DJ, Powell-Dunford N, Mazuchowski EL, Howard JT, Kotwal RS, et al. Association of prehospital blood product transfusion during medical evacuation of combat casualties in Afghanistan with acute and 30-day survival. JAMA. 2017;24(16):1581–91. 10.1001/jama.2017.15097. 10.1001/jama.2017.15097 PMC5818807 29067429

[16] O’Reilly DJ, Morrison JJ, Jansen JO, Apodaca AN, Rasmussen TE, Midwinter MJ. Prehospital blood transfusion in the en route management of severe combat trauma: a matched cohort study. J Trauma Acute Care Surg. 2014;77(3 Suppl 2):S114-20. 10.1097/TA.0000000000000328. 25159344 10.1097/TA.0000000000000328

[17] Holcomb JB, Swartz MD, DeSantis SM, Greene TJ, Fox EE, Stein DM, et al. Multicenter observational prehospital resuscitation on helicopter study. J Trauma Acute Care Surg. 2017;83(1 Suppl 1):S83–91. 10.1097/TA.0000000000001484. 28383476 10.1097/TA.0000000000001484 PMC5562146

[18] Kim BD, Zielinski MD, Jenkins DH, Schiller HJ, Berns KS, Zietlow SP. The effects of prehospital plasma on patients with injury: a prehospital plasma resuscitation. J Trauma Acute Care Surg. 2012;73(2 Suppl 1):S49–53. 10.1097/TA.0b013e31826060ff. 22847094 10.1097/TA.0b013e31826060ff

[19] Moore HB, Moore EE, Chapman MP, McVaney K, Bryskiewicz G, Blechar R, Chin T, Burlew CC, Pieracci F, West FB, Fleming CD, Ghasabyan A, Chandler J, Silliman CC, Banerjee A, Sauaia A. Plasma-first resuscitation to treat haemorrhagic shock during emergency ground transportation in an urban area: a randomised trial. Lancet. 2018;392(10144):283–91. 10.1016/S0140-6736(18)31553-8. 30032977 10.1016/S0140-6736(18)31553-8 PMC6284829

[20] Rijnhout TWH, Wever KE, Marinus RHAR, Hoogerwerf N, Geeraedts LMG Jr, Tan ECTH. Is prehospital blood transfusion effective and safe in haemorrhagic trauma patients? A systematic review and meta-analysis. Injury. 2019;50(5):1017–27. 10.1016/j.injury.2019.03.033. 30928164 10.1016/j.injury.2019.03.033

[21] Jackson BP, Sperry JL, Yazer MH. Prehospital plasma transfusion: what does the literature show? Transfus Med Hemother. 2021;48(6):358–65. 10.1159/000519627. 35082567 10.1159/000519627 PMC8740110

[22] AlJoaib NA, AlGhamdi FA, Ghafoor A, AlAnazi FZ, Maghraby NH. A systematic review and meta-analysis of prehospital plasma administration for hemorrhagic shock. J Emerg Trauma Shock. 2024;17(3):136–41. 10.4103/jets.jets_124_23. 39552833 10.4103/jets.jets_124_23 PMC11563230

[23] Abuelazm M, Rezq H, Mahmoud A, Tanashat M, Salah A, Saleh O, Morsi S, Abdelazeem B. The efficacy and safety of prehospital plasma in patients at risk for hemorrhagic shock: an updated systematic review and meta-analysis of randomized controlled trials. Eur J Trauma Emerg Surg. 2024;50(6):2697–707. 10.1007/s00068-024-02461-7. 38367091 10.1007/s00068-024-02461-7 PMC11666795

[24] Mok G, Hoang R, Khan MW, Pannell D, Peng H, Tien H, et al. Freeze-dried plasma for major trauma - systematic review and meta-analysis. J Trauma Acute Care Surg. 2021;90(3):589–602. 10.1097/TA.0000000000003012. 33507025 10.1097/TA.0000000000003012 PMC7899224

[25] Coccolini F, Pizzilli G, Corbella D, Sartelli M, Agnoletti V, Agostini V, et al. Prehospital plasma in haemorrhagic shock management: current opinion and meta-analysis of randomized trials. World J Emerg Surg. 2019;14(1):6. 10.1186/s13017-019-0226-5. 30815028 10.1186/s13017-019-0226-5 PMC6377767

[26] Wan X, Wang W, Liu J, Tong T. Estimating the sample mean and standard deviation from the sample size, median, range and/or interquartile range. BMC Med Res Methodol. 2014;14:135. 10.1186/1471-2288-14-135. 25524443 10.1186/1471-2288-14-135 PMC4383202

[27] Jost D, Lemoine S, Lemoine F, Derkenne C, Beaume S, Lanoë V, et al. Prehospital lyophilized plasma transfusion for trauma-induced coagulopathy in patients at risk for hemorrhagic shock: a randomized clinical trial. JAMA Netw Open. 2022;5(7):e2223619. 10.1001/jamanetworkopen.2022.23619. 35881397 10.1001/jamanetworkopen.2022.23619 PMC9327575

[28] Mitra B, Meadley B, Bernard S, Maegele M, Gruen RL, Bradley O, et al. Prehospital freeze-dried plasma for critical bleeding after trauma: a pilot randomized controlled trial. Acad Emerg Med. 2023;30(10):1013–9. 10.1111/acem.14745. 37103482 10.1111/acem.14745 PMC10946458

[29] Tucker H, Brohi K, Tan J, Aylwin C, Bloomer R, Cardigan R, et al. Association of red blood cells and plasma transfusion versus red blood cell transfusion only with survival for treatment of major traumatic hemorrhage in prehospital setting in England: a multicenter study. Crit Care. 2023;27(1):25. 10.1186/s13054-022-04279-4. 36650557 10.1186/s13054-022-04279-4 PMC9847037

[30] Shlaifer A, Siman-Tov M, Radomislensky I, Peleg K, Klein Y, Glassberg E, et al. The impact of prehospital administration of freeze-dried plasma on casualty outcome. J Trauma Acute Care Surg. 2019;86(1):108–15. 10.1097/TA.0000000000002094. 30358770 10.1097/TA.0000000000002094

[31] Oakeshott JE, Griggs JE, Wareham GM, Lyon RM, Kent Surrey Sussex Air Ambulance Trust. Feasibility of prehospital freeze-dried plasma administration in a UK Helicopter Emergency Medical Service. Eur J Emerg Med. 2019;26(5):373–8. 10.1097/MEJ.0000000000000585. 30531322 10.1097/MEJ.0000000000000585

[32] Clements TW, Van Gent JM, Krzyzaniak A, Campbell B, Carroll A, Mericle M, Sise M, Peck KA, Cotton BA. The effect of prehospital blood products on unexpected survival: A multi-institution study. J Trauma Acute Care Surg. 2025 May 2. 10.1097/TA.0000000000004641 10.1097/TA.0000000000004641 40312787

[33] Hamed AA, Shuib SM, Elhusein AM, Fadlalmola HA, Higazy OA, Mohammed IH, et al. Efficacy and safety of prehospital blood transfusion in traumatized patients: a systematic review and meta-analysis. Prehosp Disaster Med. 2024. 10.1017/S1049023X24000621. 39676718 10.1017/S1049023X24000621

[34] Jenkins DH, Rappold JF, Badloe JF, Berséus O, Blackbourne L, Brohi KH, et al. Trauma hemostasis and oxygenation research position paper on remote damage control resuscitation: definitions, current practice, and knowledge gaps. Shock. 2014;41(1):3–12. 10.1097/SHK.0000000000000140. 24430539 10.1097/SHK.0000000000000140 PMC4309265

[35] van Turenhout EC, Bossers SM, Loer SA, Giannakopoulos GF, Schwarte LA, Schober P. Prehospital transfusion of red blood cells. Part 2: a systematic review of treatment effects on outcomes. Transfus Med. 2020;30(2):106–33. 10.1111/tme.12659. 31903684 10.1111/tme.12659 PMC7317762

[36] Bodnar D, Rashford S, Williams S, Enraght-Moony E, Parker L, Clarke B. The feasibility of civilian prehospital trauma teams carrying and administering packed red blood cells. Emerg Med J. 2014;31(2):93–5. 10.1136/emermed-2012-201969. 23264606 10.1136/emermed-2012-201969

[37] Reitz KM, Moore HB, Guyette FX, Sauaia A, Pusateri AE, Moore EE, Hassoune A, Chapman MP, Daley BJ, Miller RS, Harbrecht BG, Claridge JA, Phelan HA, Brown JB, Zuckerbraun BS, Neal MD, Yazer MH, Sperry JL. Prehospital plasma in injured patients is associated with survival principally in blunt injury: results from two randomized prehospital plasma trials. J Trauma Acute Care Surg. 2020;88(1):33–41. 10.1097/TA.0000000000002485. 31524836 10.1097/TA.0000000000002485 PMC6923541

[38] Smith IM, James RH, Dretzke J, Midwinter MJ. Prehospital blood product resuscitation for trauma: a systematic review. Shock. 2016;46(1):3–16. 10.1097/SHK.0000000000000569. 26825635 10.1097/SHK.0000000000000569 PMC4933578

[39] Fenger-Eriksen C, Fries D, David JS, Bouzat P, Lance MD, Grottke O, et al. Prehospital plasma transfusion: a valuable coagulation support or an expensive fluid therapy? Crit Care. 2019;23(1):238. 10.1186/s13054-019-2524-4. 31262332 10.1186/s13054-019-2524-4 PMC6604317

[40] Hernandez MC, Thiels CA, Aho JM, Habermann EB, Zielinski MD, Stubbs JA, Jenkins DH, Zietlow SP. Prehospital plasma resuscitation associated with improved neurologic outcomes after traumatic brain injury. J Trauma Acute Care Surg. 2017;83(3):398–405. 10.1097/TA.0000000000001581. 28538641 10.1097/TA.0000000000001581 PMC5653265

[41] Williamson PR, Altman DG, Blazeby JM, Clarke M, Devane D, Gargon E, Tugwell P. Developing core outcome sets for clinical trials: issues to consider. Trials. 2012;13:132. 10.1186/1745-6215-13-132. PMID: 22867278; PMCID: PMC3472231. 22867278 10.1186/1745-6215-13-132 PMC3472231

[42] Lossius HM, Langhelle A, Søreide E, Pillgram-Larsen J, Lossius TA, Laake P, et al. Reporting data following major trauma and analysing factors associated with outcome using the new Utstein style recommendations. Resuscitation. 2001;50(3):263–72. 10.1016/s0300-9572(01)00361-6. 11719155 10.1016/s0300-9572(01)00361-6

