# Supplementary material for: Prehospital plasma transfusion versus standard of care following traumatic injury: a review of the systematic reviews and a meta-analysis
Source: Eur J Trauma Emerg Surg. 2025 Nov 27;51(1):354. doi: 10.1007/s00068-025-03033-z (PMC12660323; doi:10.1007/s00068-025-03033-z)
Supplement: Supplementary file 1 — Supplementary Material 1 (DOCX 26.7 KB) [file 68_2025_3033_MOESM1_ESM.docx]

| **Suppl table 1**: MeSH terms used for databases | |
| --- | --- |
| Database | Search strategy (2012–2024) |
| Pubmed | (("prehospital plasma"[All Fields] OR "plasma resuscitation"[All Fields]  OR "prehospital blood components"[All Fields] OR "emergency transfusion"[All Fields]  OR "trauma hemorrhage management"[All Fields]  OR "lyophilized plasma"[All Fields] OR "freeze-dried plasma"[All Fields]  OR "LyoPlas"[All Fields] OR "FlyPlas"[All Fields]  OR "thawed fresh frozen plasma"[All Fields])  AND  ("trauma"[All Fields] OR "hemorrhagic shock"[All Fields]))  AND ("2012/01/01"[Date - Publication] : "2024/12/31"[Date - Publication]) |
| MEDLINE (Ovid) | (prehospital plasma OR plasma resuscitation OR prehospital blood components OR emergency transfusion OR trauma hemorrhage management OR lyophilized plasma OR freeze-dried plasma OR LyoPlas OR FlyPlas OR thawed fresh frozen plasma).mp. AND (trauma OR hemorrhagic shock).mp. Limit to yr="2012 -Current". |
| EMBASE | ('prehospital plasma':ab,ti OR 'plasma resuscitation':ab,ti OR 'prehospital blood component*':ab,ti OR 'emergency transfusion':ab,ti OR 'trauma hemorrhage management':ab,ti OR 'lyophilized plasma':ab,ti OR 'freeze dried plasma':ab,ti OR 'LyoPlas':ab,ti OR 'FlyPlas':ab,ti OR 'thawed fresh frozen plasma':ab,ti) AND ('trauma'/exp OR 'hemorrhagic shock'/exp) AND [2012–2024]/py |
| Cochrane Library | (prehospital plasma OR ("prehospital" AND (plasma resuscitation OR blood components OR lyophilized plasma OR freeze-dried plasma OR thawed fresh frozen plasma)) OR LyoPlas OR FlyPlas OR trauma hemorrhage management)  AND (trauma OR hemorrhagic shock)  with Publication Year from 2012 to 2024 |

| **Suppl table 2: The Newcastle-Ottawa Scale for the observational studies** | | | | |
| --- | --- | --- | --- | --- |
| **Study** | **Selection** | **Comparability** | **Outcome** | **Score** |
| Henrisken 2016⃰ | 3 | 2 | 3 | 8/9 |
| Shackelford 2017⃰ | 4 | 2 | 3 | 9/9 |
| Holocomb 2017 | 4 | 2 | 3 | 9/9 |
| Kim 2012 | 3 | 0 | 3 | 6/9 |
| Tucker 2023 | 4 | 2 | 3 | 9/9 |
| Shlaifer 2018 | 4 | 2 | 3 | 9/9 |
| Oakeshott 2018⃰ | 4 | 0 | 3 | 7/9 |
| ⃰Included in the systematic review, not in the meta-analysis | | | | |

| **Supl table 3: Cochrane ROB2 assessment of Randomized controlled trials** | | | | | | |
| --- | --- | --- | --- | --- | --- | --- |
|  | **Risk of bias domains** | | | | |  |
|  | **D1** | **D2*** | **D3** | **D4** | **D5** | **Overall** |
| **Mitra 2023** | Low | Some concerns | Low | Low | Low | Some concerns |
| **Jost 2022** | Low | Some concerns | Low | Low | Low | Some concerns |
| **Moore 2018** | Low | Some concerns | Low | Low | Low | Some concerns |
| **Sperry 2018** | Low | Some concerns | Low | Low | Low | Some concerns |
| **Crombie 2023** | Low | Some concerns | Low | Low | Low | Some concerns |
| ROB: Risk of bias, D1: Risk of bias arising from the randomization process; D2: Risk of bias due to deviations from the intended interventions; D3: Missing outcome data; D4: Risk of bias in measurement of the outcome; D5: Risk of bias in selection of the reported result. *All studies were open label, no deviations from the protocol were reported. Therefore, we did not downgrade the D2 to high-risk. | | | | | | |

| Suppl table 4. GRADE assessment | | | |
| --- | --- | --- | --- |
| **GRADE assessment for 24-hour mortality** | | | |
| **Criteria** | **Assessment** | **Effect on Quality** | **Resulting Quality of Evidence** |
| Starting Quality of Evidence | Moderate (Reason: Mixed evidence from 3 RCTs and 1 influential prospective non-RCT). |  | Moderate |
| 1. Risk of Bias | Not Serious. Sensitivity analysis includes three RCTs (Sperry 2018, Moore 2018, Jost 2022) with "some concerns" (not deemed serious for this outcome) and one good quality non-RCT (Tucker 2023). | No downgrade | Moderate |
| 2. Inconsistency (Heterogeneity) | Not serious. I² = 14% (Figure 3b), indicating low statistical heterogeneity. | No downgrade | Moderate |
| 3. Indirectness | Serious. While three RCTs focused on adults, the influential Tucker 2023 study included "all ages," introducing indirectness to the specific question about adult trauma patients. | Downgrade by 1 | Low |
| 4. Imprecision | Not serious. The 95% CI for the RR is 0.61 to 0.94. It does not cross the line of no effect (RR=1.0) and suggests a potentially clinically important benefit. (Number of participants: 2743). | No downgrade | Low |
| 5. Publication Bias | Not downgraded. The systematic review reports that publication bias was assessed (Funnel plot Figure 2b, Egger's p=0.08 for sensitivity analysis) and not found to be significant. | No downgrade | Low |
| Other factors (e.g., large effect, dose-response, opposing plausible confounding) | None identified that would upgrade the quality. The effect size (RR 0.76) is not considered large enough (e.g., RR < 0.5 or > 2.0) to warrant upgrading on its own. | N/A | Low ⊕⊕⊖⊖ |
| **GRADE assessment for 30 Day mortality** | | | |
| Starting Quality of Evidence | Moderate (Reason: Mixed evidence from 3 RCTs and 1 influential prospective non-RCT). |  | Moderate |
| 1. Risk of Bias | No serious limitations. The "Some Concerns" in RCTs (primarily due to unavoidable open-label design) were judged by the study authors as not leading to significant bias for the mortality outcome. | No downgrade | Moderate |
| 2. Inconsistency (Heterogeneity) | Not serious. I² = 28.63% for Figure 3d, indicating low to moderate heterogeneity. | No downgrade | Moderate |
| 3. Indirectness | Serious. The Tucker 2023 study (weighted at 56.4% in Figure 3d) included "All ages," while the research question focuses on adult trauma patients. | Downgrade by 1 | Low |
| 4. Imprecision | Serious. The 95% CI for the RR (0.71 to 1.08) is wide and crosses the line of no effect (RR=1.0), including both potentially important benefit and no effect/slight harm. | Downgrade by 1 | Very Low |
| 5. Publication Bias | Not suspected. Formal tests reported in the manuscript did not suggest significant publication bias. | No downgrade | Very Low |
| Other factors (e.g., large effect, dose-response, opposing plausible confounding) | None identified that would upgrade the quality. | N/A | Very Low ⊕⊖⊖⊖ |
| **GRADE assessment 24-hour use of RBC** | | | |
| Starting Quality of Evidence | Moderate (Reason: Based on 3 RCTs and 1 prospective non-RCT study for this specific analysis). |  | Moderate |
| 1. Risk of Bias | Serious. For this outcome (RBC units), the volume component of the plasma intervention could directly influence hemodynamic stability and decisions to transfuse RBCs, potentially confounding the assessment of plasma's specific hemostatic effects. This is in addition to "some concerns" in RCTs' open-label design. | Downgrade by 1 | Low |
| 2. Inconsistency (Heterogeneity) | Serious. I² = 72.33%, indicating substantial statistical heterogeneity across studies. | Downgrade by 1 | Very Low |
| 3. Indirectness | Serious. The Tucker 2023 study (weighted at 34.4% in this analysis) included "All ages," while the research question focuses on adult trauma patients. This introduces population indirectness. | Downgrade by 1 | Very Low |
| 4. Imprecision | Not serious. The 95% CI for the Mean Difference (-2.18 to -0.19 units) does not cross the line of no effect (MD=0). Both ends of the CI suggest a reduction in RBC use, ranging from a small to a moderate effect. | No downgrade | Very Low |
| 5. Publication Bias | Not downgraded. The systematic review's overall assessment did not suggest significant publication bias. | No downgrade | Very Low |
| Other factors (e.g., large effect, dose-response, opposing plausible confounding) | None identified that would upgrade the quality. | N/A | Very Low ⊕⊖⊖⊖ |
| * The certainty of evidence was initially started as high because most included studies were RCTs, but it was downgraded by one level due to the inclusion of a non-RCT and considered moderate. | | | |

| **Suppl** table 5: Funding source and their role in the included studies | | | |
| --- | --- | --- | --- |
| **Study (Author, Year)** | **Journal** | **Funding Source** | **Role of the Funder** |
| **Shackelford et al., 2017** | JAMA | US Army & US Dept. of Energy via USAMRMC | No role |
| **Sperry et al., 2018 (PAMPer)** | N Engl J Med | US Army Medical Research (W81XWH-12-2-0023) | No role |
| **Crombie et al., 2022 (RePHILL)** | Lancet Haematol | NIHR – Efficacy and Mechanism Evaluation Programme | No role |
| **Holcomb et al., 2017 (PROHS)** | J Trauma Acute Care Surg | US National Heart, Lung, and Blood Institute & US Dept. of Defense | No role |
| **Oakeshott et al., 2018** | Not specified | Not reported | Not reported |
| **Jost et al., 2022 (PREHO-PLYO)** | JAMA Surg | French Defence Central Health Service | No role |
| **Mitra et al., 2023** | Transfusion | National Blood Authority & NHMRC (Australia) | No role |
| **Shlaifer et al., 2018** | J Trauma Acute Care Surg | Israel Defense Forces & Israeli Ministry of Defense | Not explicitly stated |
| **Tucker et al., 2023** | Critical Care | London Air Ambulance, Barts Charity, NHS Blood and Transplant | Not explicitly stated |
| **Kim et al., 2012** | J Trauma Acute Care Surg | Not reported | Not reported |
| **Moore et al., 2018 (COMBAT)** | Lancet | US Dept. of Defense, NIH, Haemonetics, Instrumentation Laboratory | No role |
| **Henriksen et al., 2016** | Scand J Trauma Resusc Emerg Med | Not reported | Not reported |
