# Supplementary material for: Prehospital plasma transfusion versus standard of care following traumatic injury: a review of the systematic reviews and a meta-analysis
Source: Eur J Trauma Emerg Surg. 2025 Nov 27;51(1):354. doi: 10.1007/s00068-025-03033-z (PMC12660323; doi:10.1007/s00068-025-03033-z)
Supplement: Supplementary file 2 — Supplementary Material 2 (DOCX 122 KB) [file 68_2025_3033_MOESM2_ESM.docx]

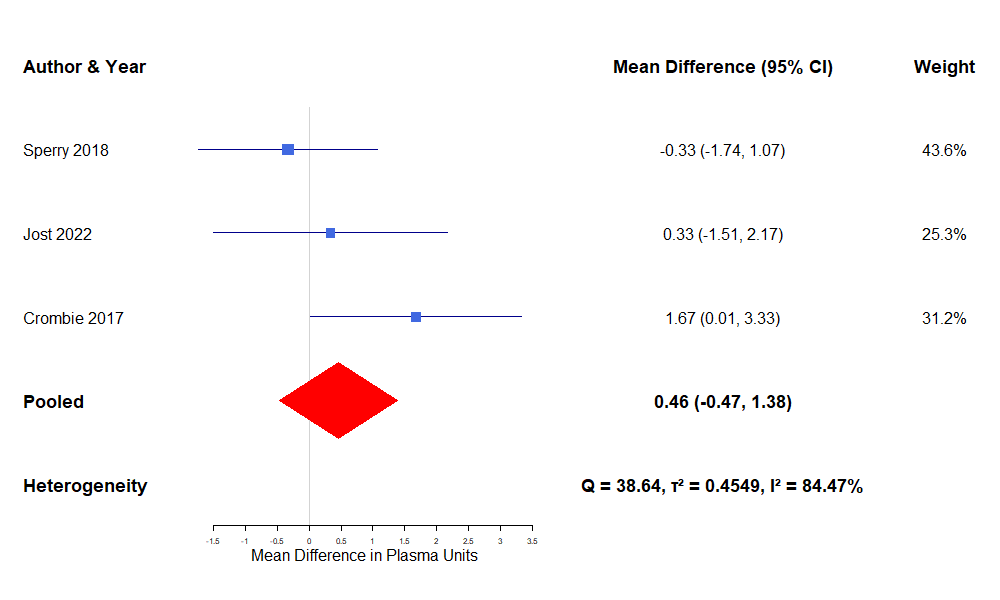


Suppl 1: forest plot for 24-hour Plasma transfusion used


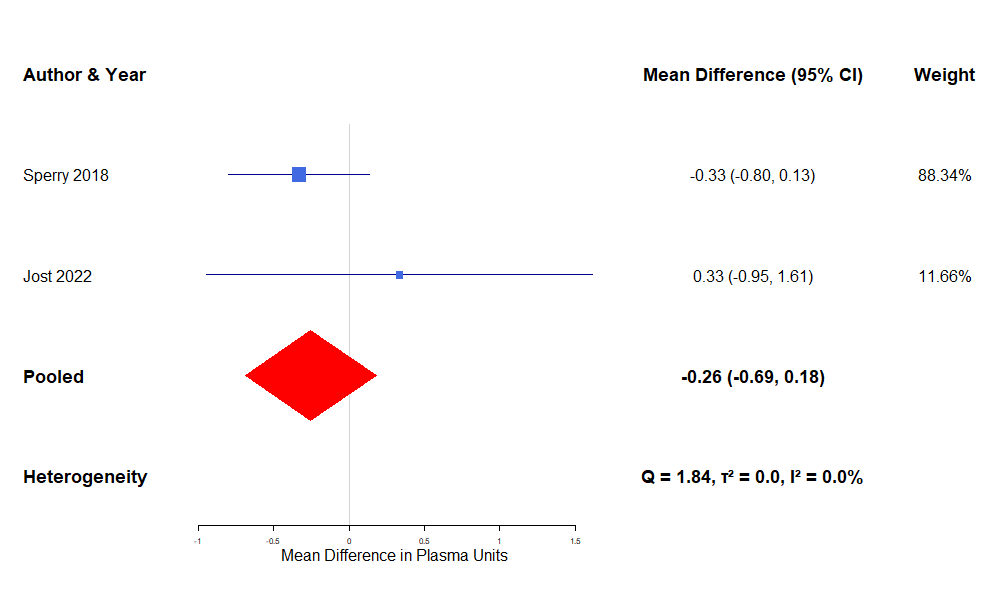


Suppl 1b : forest plot for 24-hour Plasma transfusion used- sensitivity analysis


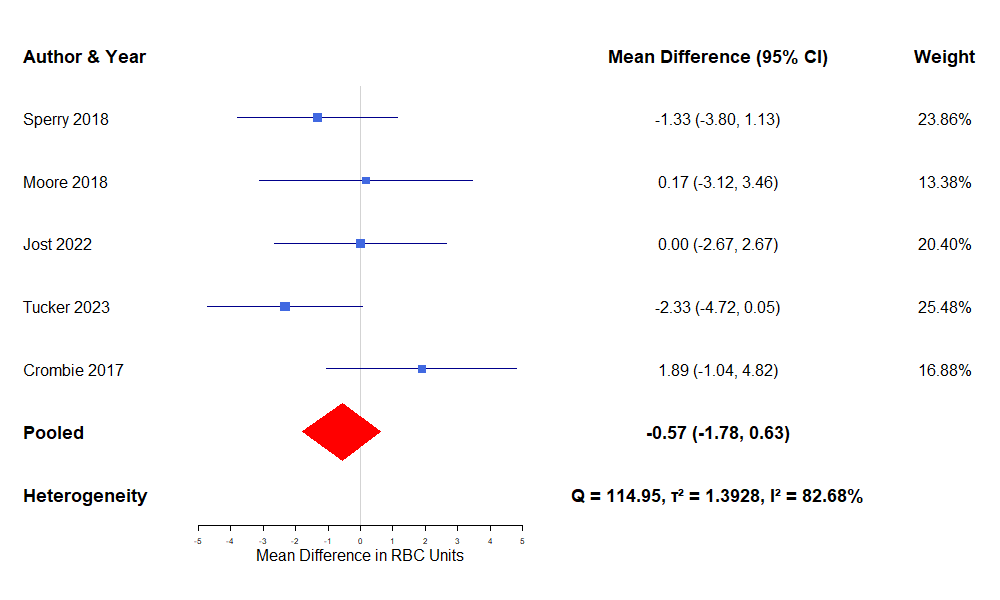


Suppl 2: forest plot for 24-hour RBC transfusion


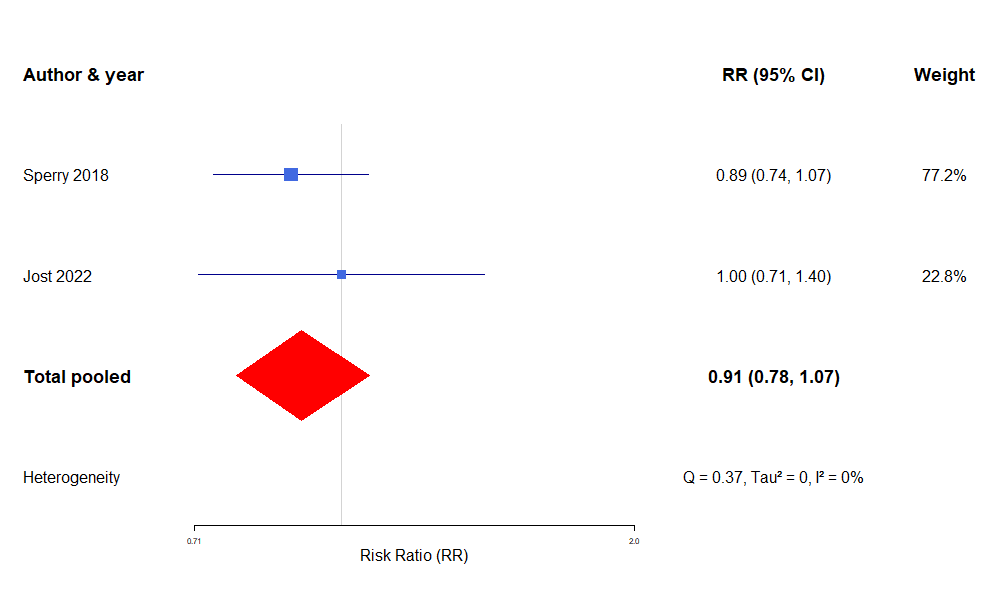


Suppl 3: Forest plot for Vasopressors received


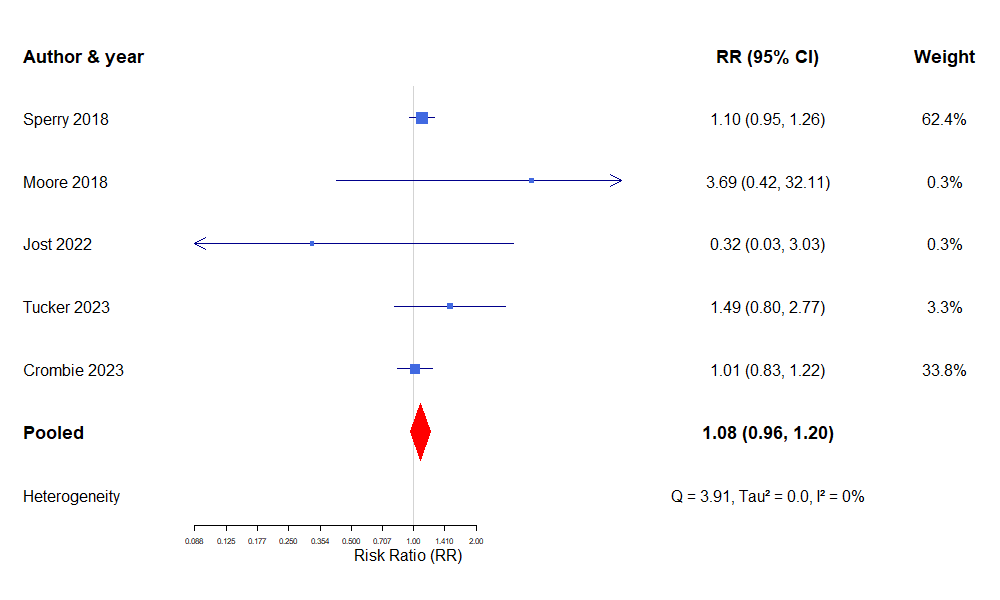


Suppl 4: Forest plot for MOF


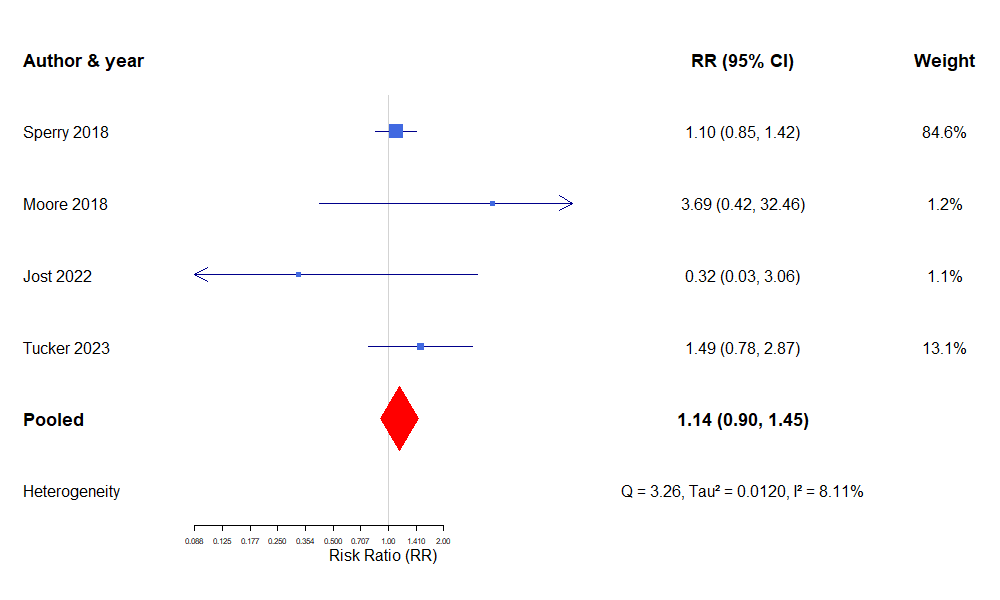


Suppl 4 b: Forest plot for MOF- sensitivity analysis


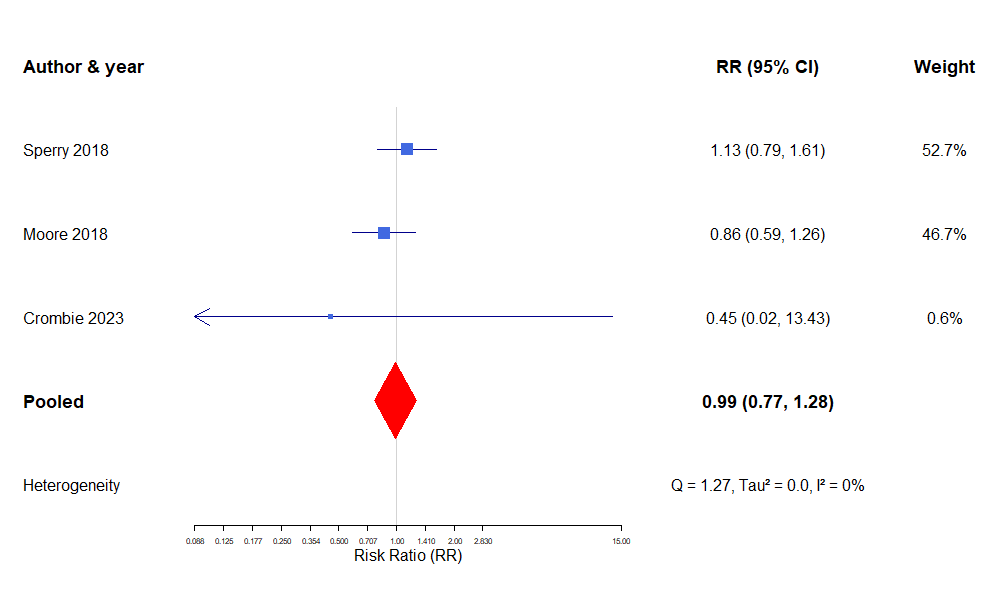


Suppl 5: Forest plot for Acute lung injury


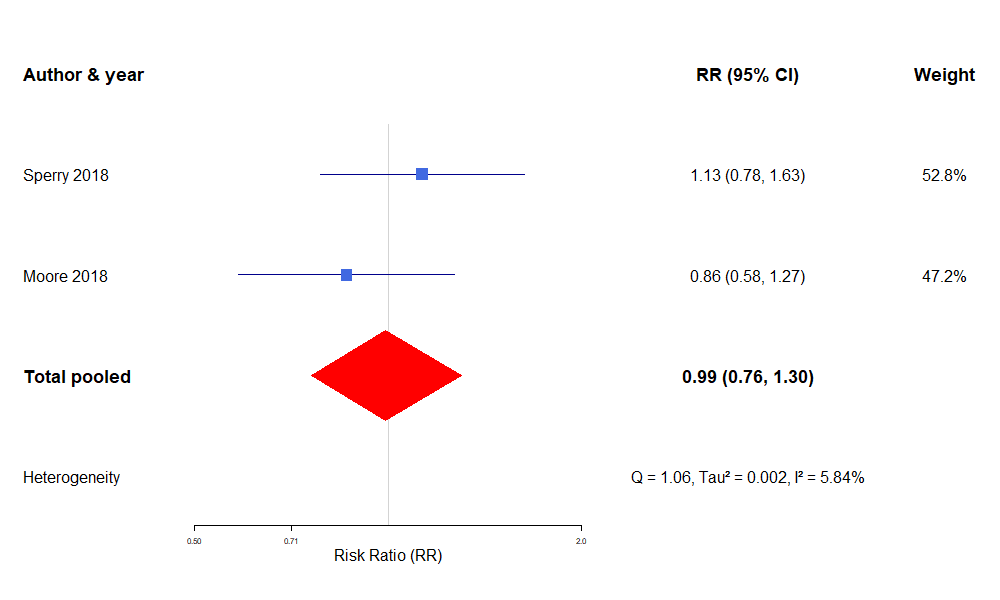


Suppl 5 b: Forest plot for Acute lung injury- sensitivity analysis


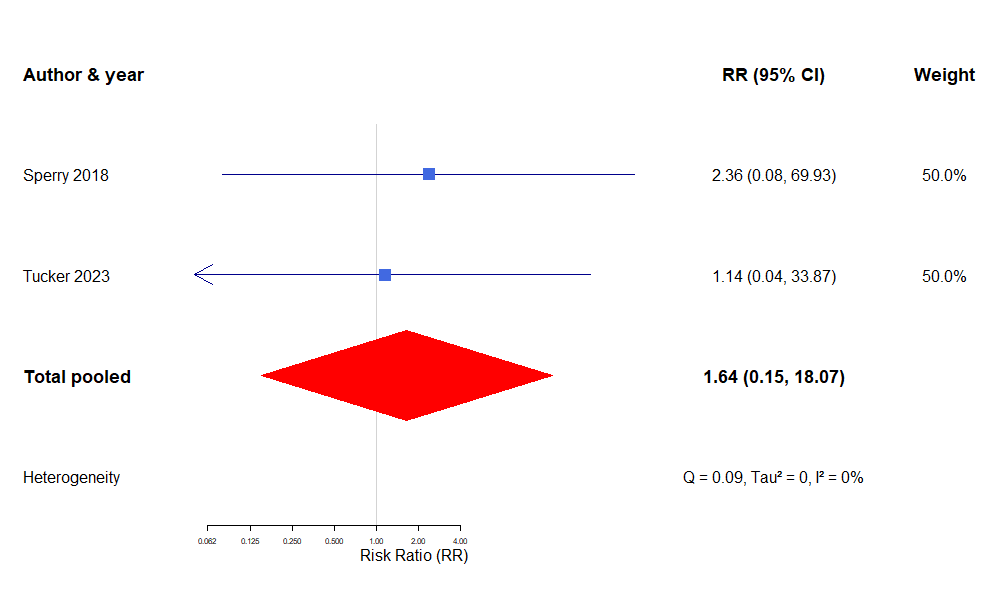


Suppl 6: Forest plot for transfusion reaction


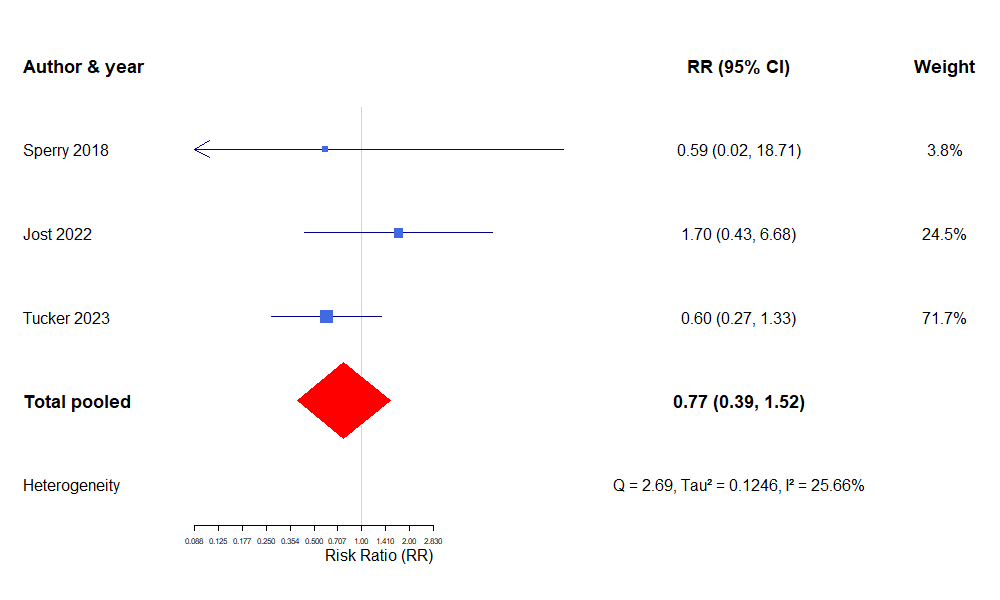


Suppl 7: Forest plot for sepsis
